# Convergent evolution of the UbiA prenyltransferase family underlies the independent acquisition of furanocoumarins in plants

**DOI:** 10.1111/nph.16277

**Published:** 2019-11-19

**Authors:** Ryosuke Munakata, Sakihito Kitajima, Andréïna Nuttens, Kanade Tatsumi, Tomoya Takemura, Takuji Ichino, Gianni Galati, Sonia Vautrin, Hélène Bergès, Jérémy Grosjean, Frédéric Bourgaud, Akifumi Sugiyama, Alain Hehn, Kazufumi Yazaki

**Affiliations:** ^1^ Laboratory of Plant Gene Expression Research Institute for Sustainable Humanosphere Kyoto University Uji Kyoto 611‐0011 Japan; ^2^ Université de Lorraine INRA, LAE F54000 Nancy France; ^3^ Department of Applied Biology Kyoto Institute of Technology Matsugasaki Sakyo‐ku Kyoto 606‐8585 Japan; ^4^ The Center for Advanced Insect Research Promotion Kyoto Institute of Technology Matsugasaki Sakyo‐ku Kyoto 606‐8585 Japan; ^5^ Centre National de Ressources Genomiques Vegetales – INRA 24 Chemin de Borde Rouge Auzeville CS 52627 31326 Castanet Tolosan Cedex France; ^6^ Plant Advanced Technologies – PAT 19 Avenue de la forêt de Haye 54500 Vandoeuvre France

**Keywords:** convergent evolution, fig (*Ficus carica*), furanocoumarin, latex, Moraceae, prenyltransferase, UbiA superfamily

## Abstract

- Furanocoumarins (FCs) are plant‐specialized metabolites with potent allelochemical properties. The distribution of FCs is scattered with a chemotaxonomical tendency towards four distant families with highly similar FC pathways. The mechanism by which this pathway emerged and spread in plants has not been elucidated.
- Furanocoumarin biosynthesis was investigated in *Ficus carica* (fig, Moraceae), focusing on the first committed reaction catalysed by an umbelliferone dimethylallyltransferase (UDT). Comparative RNA‐seq analysis among latexes of different fig organs led to the identification of a *UDT*. The phylogenetic relationship of this *UDT* to previously reported Apiaceae *UDTs* was evaluated.
- The expression pattern of *F. carica prenyltransferase 1* (*FcPT1*) was related to the FC contents in different latexes. Enzymatic characterization demonstrated that one of the main functions of FcPT1 is UDT activity. Phylogenetic analysis suggested that FcPT1 and Apiaceae UDTs are derived from distinct ancestors, although they both belong to the UbiA superfamily. These findings are supported by significant differences in the related gene structures.
- This report describes the identification of *FcPT1* involved in FC biosynthesis in fig and provides new insights into multiple origins of the FC pathway and, more broadly, into the adaptation of plants to their environments.

## Introduction

Furanocoumarins (FCs) are a group of plant‐specialized metabolites, consisting of over 200 derivatives to date. FCs have been classified into two distinct subgroups, linear and angular, based on the positions of the furan ring associated with the coumarin core structure (Seiger, 1998; Bourgaud *et al*., 2006, 2014). These molecules contribute to plant chemical defences, mainly against biotic stresses, such as herbivores and pathogens (Bourgaud *et al*., 2014), and are also a key element in the arms race between Apiaceae and Lepidopteran insects (Berenbaum & Feeny, 1981). FCs show a scattered distribution in angiosperms, with a chemotaxonomical tendency towards four distant plant families: Apiaceae, Fabaceae, Moraceae and Rutaceae (Supporting Information Fig. S1) (Murray *et al*., 1982). These families include medicinally and agronomically important species, such as Apiaceae herbs and citrus plants, in which these metabolites are generally considered as pharmaceutical and toxic constituents (Bourgaud *et al*., 2014; Dugrand‐Judek *et al*., 2015).

**Figure 1:**
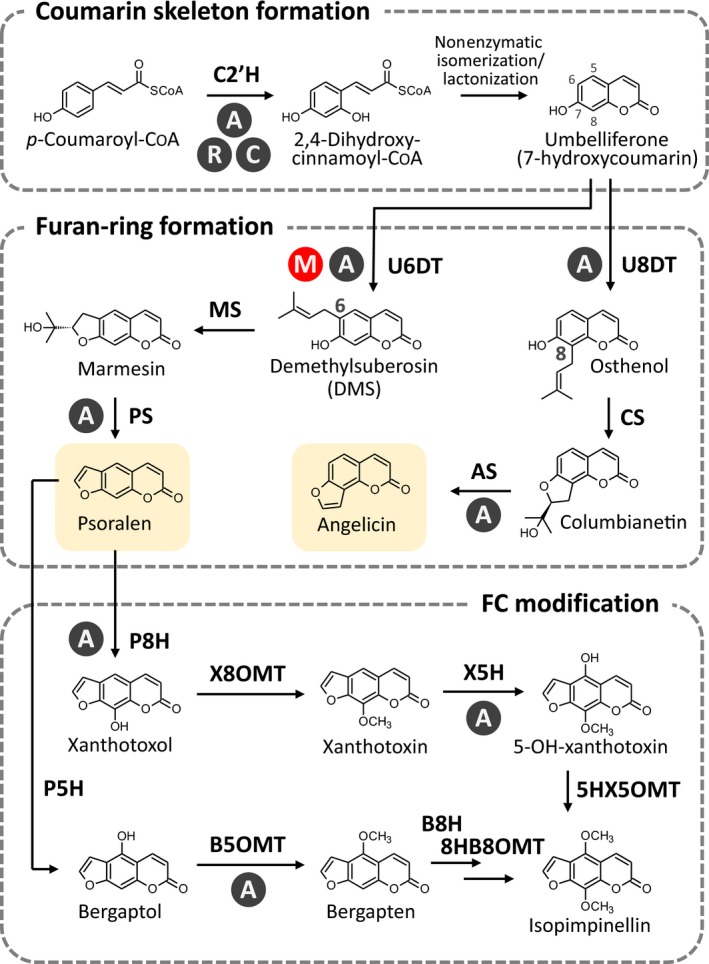
Simple coumarin and furanocoumarin (FC) biosynthetic pathways in plants. The FC pathways from *p*‐coumaroyl‐CoA to isopimpinellin (linear‐type) and angelicin (angular‐type) via umbelliferone, a simple coumarin molecule serving as the common precursor for both FC types, are shown. Psoralen and angelicin, highlighted in pale yellow, are the core skeleton structures of linear and angular FCs, respectively. Genetically characterized reaction steps are marked according to their botanical origins: A, Apiaceae; C, Convolvulaceae, M, Moraceae (in this study); R, Rutaceae. Abbreviations for enzymes are as follows: C2′H, *p*‐coumaroyl CoA 2′‐hydroxylase; U6DT, umbelliferone 6‐dimethylallyltransferase; U8DT, umbelliferone 8‐dimethylallyltransferase; MS, marmesin synthase; CS, columbianetin synthase; PS, psoralen synthase; AS, angelicin synthase; P5H, psoralen 5‐hydroxylase; P8H, psoralen 8‐hydroxylase; B5OMT, bergaptol 5‐*O*‐methyltransferase; X8OMT, xanthotoxol 8‐*O*‐methyltransferase; B8H, bergapten 8‐hydroxylase; X5H, xanthotoxin 5‐hydroxyase; 8HB8OMT, 8‐hydroxybergapten 8‐*O*‐methyltransferae; 5HX5OMT, 5‐hydroxyxanthotoxin 5‐*O*‐methyltransferase.

The FC biosynthetic pathway was initially investigated by feeding experiments with radiolabelled chemicals and isolation of intermediate compounds (Brown & Steck, 1973; Murray *et al*., 1982). These studies indicated that the major FC‐producing families synthesize psoralen, the linear FC core structure, through similar pathways, although this route has not been fully assessed in Fabaceae. In the linear FC pathway, umbelliferone, a common coumarin derivative in angiosperms, is first dimethylallylated to yield demethylsuberosin (DMS, 6‐dimethylallylumbelliferone), which is subsequently converted to psoralen via marmesin (Fig. 1). Angelicin, representing the angular FC backbone, is also synthesized from umbelliferone through an analogous pathway (Fig. 1). Angular FCs have a more restricted taxonomical distribution than linear FCs, with most angular FCs being detected in Apiaceae species (Berenbaum & Feeny, 1981). Moreover, angular FCs are thought to have appeared later than linear FCs in the course of Apiaceae evolution (Ma *et al*., 1994; Larbat *et al*., 2009). The intermediates in angular FC biosynthesis, osthenol (8‐dimethylallylumbelliferone) and columbianetin, have also been isolated from a Rutaceae species that accumulates angelicin (Fig. 1) (Filippini *et al*., 1998). Hence, like linear FCs, the biosynthetic reaction steps required for various angular FCs among different unrelated plant taxa may be identical. The common routes leading to the synthesis of FC core structures among taxonomically distant plant families suggest two alternative hypotheses for the emergence of this metabolite group: the development of the pathway in a common ancestor followed by its loss in many descendant taxa; or the independent emergence of a common FC biosynthetic pathway.

Most studies assessing the molecular characterization of FC biosynthesis were performed in Apiaceae species (Bourgaud *et al*., 2006, 2014), which led to the identification of a series of FC biosynthetic genes. Cytochrome P450 monooxygenases (P450s) were described as being responsible for furan‐ring formation; genes encoding these enzymes include *CYP71AJ1*, which encodes a psoralen synthase (PS) (Larbat *et al*., 2007), and *CYP71AJ4*, which encodes an angelicin synthase (AS) (Larbat *et al*., 2009). Other P450 genes involved in subsequent modifications of the FC cores include *CYP71AZ4*, which encodes a psoralen 8‐hydroxylase (Krieger *et al*., 2018), and *CYP71AZ1/6*, which encodes a xanthotoxin 5‐hydroxylase (Krieger *et al*., 2018). Bergaptol 5‐*O*‐methyltransferases have also been reported to belong to the SABATH superfamily (Hehmann *et al*., 2004; Ishikawa *et al*., 2009; Zhao *et al*., 2016) (Fig. 1).

Particular attention has focused on the initial step in the FC pathway, catalysed by an umbelliferone dimethylallyltransferase (UDT). The regiospecific transfer performed by this enzyme of a dimethylallyl moiety to the C6 or C8 of umbelliferone (U6DT or U8DT reaction) enables entry into either the linear or the angular FC pathway, respectively (Fig. 1) (Brown & Steck, 1973). Reports published in the 1970s showed that the native U6DT activity in *Ruta graveolens* (Rutaceae) was associated with chloroplast membranes and required divalent cations as cofactors (Ellis & Brown, 1974; Dhillon & Brown, 1976). Recently, several *prenyltransferase* (*PT*) genes involved in the synthesis of FCs have been identified, and their gene products have been shown to preferentially catalyse the U6DT or U8DT reaction (i.e. U6DT encoded by *Petroselinum crispum PT1* (*PcPT1*) and *Pastinaca sativa PT1* (*PsPT1*), and U8DT encoded by *P. sativa PT2* (*PsPT2*)) (Karamat *et al*., 2014; Munakata *et al*., 2016). These PTs all belong to the UbiA superfamily, a PT family of membrane‐bound proteins possessing two aspartate‐rich motifs that are conserved motifs crucial for the divalent cation‐dependent prenylation (Winkelblech *et al*., 2015). The Apiaceae UDTs are shown to be localized to the plastids. These results suggest that this first step of FC biosynthesis in different plant taxa is catalysed by the same enzyme family.

To clarify the pattern of emergence of FCs in plants, this study focused on the PT genes involved in the first step of FC biosynthesis in fig (*Ficus carica*), a Moraceous plant that accumulates a large quantity of linear FC derivatives in its laticifer cells (latexes) (Zaynoun *et al*., 1984). Recently, ‐omics resources of this species have been created (Mori *et al*., 2017; Kitajima *et al*., 2018), including comparable RNA‐seq libraries from latexes of different fig organs (fruit, petiole, and trunk) (Kitajima *et al*., 2018). Taking advantage of individual gene expression profiles in these libraries, we identified a fig *U6DT* and characterized the enzymatic properties of its encoded protein. The phylogenetic relationship of this UDT to previously reported Apiaceae UDTs was also analysed.

## Materials and Methods

### Plant materials and reagents

Latexes (Fig. S2) were collected from five individual fig trees maintained in the Center for Bioresource Field Science, Kyoto Institute of Technology, Kyoto (Japan). A standard specimen of DMS was purchased from Topharman (Shanghai, China). Phenolic substrates and prenyl diphosphates were purchased from Tokyo Chemical Industry Co., Ltd (Tokyo, Japan), Extrasynthese (Lyon, France), Herboreal Ltd (Dalkeith, UK) and Sigma‐Aldrich. Dimethylallyl diphosphate (DMAPP) was also generously provided by Dr Hirobumi Yamamoto (Toyo University, Japan) and used for preliminary analysis. Geranylgeranyl diphosphate (GGPP) was generously provided by Dr Nathalie Giglioli‐Guivarc’h (Université François‐Rabelais de Tours, France).

### Construction of an RNA‐seq library from latexes of fig fruits

An RNA‐seq library was prepared from latexes of fig fruits as previously described (Kitajima *et al*., 2018). The contig sequences used in this study are shown in Fig. S3.

### Isolation of FcPT genes and construction of plant expression plasmids

Latexes collected from fig fruits were mixed with a 10‐fold volume of TRIzol reagent (Thermo Fisher Scientific, Waltham, MA, USA) and frozen in liquid nitrogen. Total RNA was extracted as described (Kitajima *et al*., 2012), and cDNA was synthesized with SuperScript III First‐Strand Synthesis Supermix (Invitrogen). The nucleotide sequences containing the full coding sequences (CDSs) of four *FcPT* genes were amplified by PCR using KOD‐plus neo or ver.2 (Toyobo, Osaka, Japan), the synthesized cDNA pool as a template, and primer pairs for *FcPT1a* (FcPT1_5′UTR_Fw and FcPT1a_3′UTR_Rv), *FcPT1b* (FcPT1_5′UTR_Fw and FcPT1b_3′UTR_Rv), *FcPT2a* (FcPT2_5′UTR_Fw and FcPT2a_3′UTR_Rv), and *FcPT2b* (FcPT2_5′UTR_Fw and FcPT2b_3′UTR_Rv) (Table S1). The amplicons were inserted into the pGEM T‐easy vector (Promega) for sequencing.

The CDSs of *FcPTs* were further amplified by PCR using KOD‐plus neo or ver.2 and the primer pairs for *FcPT1a/b* (FcPT1_TOPO_Fw and FcPT1_TOPO_Rv) and *FcPT2a/b* (FcPT2_TOPO_Fw and FcPT2_TOPO_Rv) (Table S1), and the PCR products were subsequently inserted into the pENTR^TM^/D‐TOPO^®^ vector (Invitrogen) by directional TOPO reactions. The resulting entry vectors were subsequently introduced into the pGWB502 binary vector by LR recombination (Nakagawa *et al*., 2007), yielding pGWB502‐*FcPT* constructs possessing *P35S*‐*FcPT*‐*Tnos*. The pGWB505‐*FcPT1a/bTP* constructs containing *P35S‐FcPT1a/bTP‐synthetic green fluorescent protein* (*sGFP*)*‐Tnos* for subcellular localization analysis were constructed by the same process using the pGWB505 vector (Nakagawa *et al*., 2007) and the primer pairs for amplification of the nucleotide sequences encoding the first 72 and 70 amino acids of FcPT1a and FcPT1b, respectively (FcPT1_TOPO_Fw and FcPT1TP_Rv) (Table S1).

### Transient expression of FcPTs in *Nicotiana benthamiana* leaves and preparation of microsomes

Recombinant FcPT proteins were produced in *N. benthamiana* leaves by agroinfiltration using the pBIN61‐P19 plasmid, and microsomes were prepared from the leaves as previously described (Voinnet *et al*., 2003; Karamat *et al*., 2014), except that the leaves in this study were ground with mortar and pestle. Each microsomal fraction was suspended in 100 mM Tris‐HCl buffer containing 1.0 mM dithiothreitol and stored at −80°C. The protein concentrations of microsomes were quantified with a Qubit_2.0 fluorometer (Invitrogen) according to the manufacturer’s protocol.

### *In vitro* PT assay

A standard mixture (200 µl) containing 200 µM prenyl acceptor substrate, 200 µM prenyl donor substrate, 10 mM MgCl_2_, and microsomes as crude enzymes (0.12 µg of total proteins) was incubated at 28°C for 16 h, unless otherwise described. Enzymatic reactions were stopped by the addition of 100 µl of 3 M HCl, and phenolic compounds were extracted with ethyl acetate as previously described (Munakata *et al*., 2014).

### LC/MS analysis of enzymatic products

Reaction products were analysed using a Shimadzu Nexera ultra‐high‐performance LC‐photodiode array (UHPLC‐PDA) system (Shimadzu, Kyoto, Japan) to assess the substrate specificity of FcPT1a and the LC20A HPLC‐PDA system (Shimadzu) for other routine analyses. The UHPLC‐PDA analysis was performed essentially as described by Krieger *et al*. (2018). In the prenyl donor specificity test, acetonitrile was used instead of methanol as a solvent. For HPLC‐PDA analysis, reaction products were separated on a C18 Interchim Vintage series (LR RP18E 250 × 4.0 mm, 5 µm; Interchim, Montluçon, France) column using a programme composed of an isocratic step of 10% (v/v) solvent B (methanol with 0.1% (v/v) formic acid) in solvent A (MilliQ water with 0.1% (v/v) formic acid) over 0–3 min and the following gradient step of 10% to 99% (v/v) over 3–34 min at room temperature and a flow rate of 0.7 ml min^–1^. Reaction products were detected based on UV scans ranging from 190 to 450 nm.

Reaction products were identified using an LC‐MS/MS ‘LTQ Orbitrap’ (Thermo Fisher Scientific) system. After chromatographic separation similar to UHPLC analysis, the reaction products were ionized in electrospray ionization mode followed by detection using a mass scan ranging from *m*/*z* 80 to 800.

### Extraction and quantification of FCs from fig latexes

Fruits, petioles and trunks of fig trees were cut, and extruded latex was collected. These latexes were immediately frozen in liquid nitrogen and stored at –80°C. Following thawing, 30 mg of latex was added to 300 µl of methanol, and the samples were vortexed at 2500 rpm at room temperature for 10 min. After centrifugation at 20 400 ***g*** at room temperature for 5 min, the supernatant fraction was collected. Next, the pellet was subjected again to this extraction procedure. The two supernatant fractions were combined and dried with nitrogen gas. The extract was dissolved in 500 µl of methanol and filtered through Minisart^®^ RC4 (0.2 mm pore; Sartorius Stedim Biotech, Göttingen, Germany). FCs in latex extracts were quantified with a D‐2000 Elite HPLC System (Hitachi, Tokyo, Japan) as previously described (Munakata *et al*., 2014).

### Quantitative RT‐PCR

Total RNA pools were extracted from latexes of fig fruits, petioles and trunks, as previously described (Kitajima *et al*., 2012), and reverse‐transcribed with ReverTra Ace^®^ qPCR RT Master Mix with gDNA Remover (Toyobo). The synthesized cDNA pools were used as templates for quantitative reverse transcription polymerase chain reaction (qRT‐PCR) using Thunderbird^®^ SYBR^®^ qPCR Mix (Toyobo), the *FcPT1a/b* primer pair (FcPT1_qPCR_Fw and FcPT1_qPCR_Rv), and the primer pair for *FcActin* (FcActin_qPCR_Fw and FcActin_qPCR_Rv) as a reference gene (Ikegami *et al*., 2013) (Table S1). These PCRs were conducted under the control of CFX96 Deep Well (Bio‐Rad) using an amplification programme consisting of initial denaturation at 98°C for 2 min followed by 45 cycles of denaturation at 98°C for 10 s, annealing at 55°C for 10 s, and elongation at 68°C for 30 s. Amplification of the target sequences was confirmed by sequencing.

### Transient expression of FcPT1TP‐sGFP in *N. benthamiana* leaves and microscopic observation

The FcPT1a/bTP‐sGFP‐expression constructs were introduced into *N. benthamiana* leaves by agroinfiltration as previously described (Karamat *et al*., 2014), except that the pBIN61‐P19 vector was not used in this analysis. Forty‐eight hours later, fluorescence images of epidermal cells of the leaves were acquired using a confocal laser scanning microscope (FV3000; Olympus, Tokyo, Japan) with a 20 × 0.75 numerical aperture objective (UPLSAPO 20×; Olympus). The 488 nm line of a 20 mW diode laser and an emission filter (bandpass 500–540 nm) were used to detect the GFP fluorescence, and the 640 nm line of a 40 mW diode laser and an emission filter (bandpass 650–750 nm) were used to detect Chl autofluorescence. The pHKN29 plasmid containing *P35S‐sGFP‐Tnos* was used as a control for free sGFP (Kumagai & Kouchi, 2003). The acquired images were processed by FV31S‐SW software (Olympus).

### Sequencing of bacterial artificial chromosome (BAC) clones containing PsPT1 and PsPT2

The previously reported genomic sequence of *PsPT1* (Roselli *et al*., 2017) was registered with the NCBI in this report. A 532 nucleotide sequence corresponding to *PsPT2* was amplified from genomic DNA extracted from plant seedlings with the E.Z.N.A. Plant DNA kit (Omega Bio‐Tek, Norcross, GA, USA) using PrimeSTAR Max DNA Polymerase (Takara, Japan) and the primer pair (PsPT2_BAC_Fw and PsPT2_BAC_Rv) (Table S1). This probe was used to screen a parsnip BAC library, as described previously (Roselli *et al*., 2017).

### *In silico* analyses

Contigs belonging to the UbiA superfamily in RNA‐seq libraries of fig latexes were screened by tblastn searching using bioedit software (http://www.mbio.ncsu.edu/BioEdit/bioedit.html). Contigs homologous to *FcPT1* were obtained from the RNA‐seq library of *Ficus religiosa* leaves available in OneKP (https://sites.google.com/a/ualberta.ca/onekp/) (Johnson *et al*., 2012; Matasci *et al*., 2014; Wickett *et al*., 2014; Xie *et al*., 2014). bioedit software was also used to calculate amino acid identities among polypeptide sequences. The transmembrane regions and the transit peptides of FcPTs were predicted by tmhmm server v.2.0 (http://www.cbs.dtu.dk/services/TMHMM/) and chlorop (http://www.cbs.dtu.dk/services/ChloroP/), respectively. Phylogenetic trees were constructed based on clustalw multiple alignments using mega7 (http://www.megasoftware.net/). Genomic sequences were obtained from NCBI (https://www.ncbi.nlm.nih.gov/), phytozome v.12.1.6 (https://phytozome.jgi.doe.gov/pz/portal.html#) and MorusDB (https://morus.swu.edu.cn/).

### Statistical analyses

Statistical analyses were performed using R software (R Core Team, 2018). The apparent *K*_m_ values were calculated by a nonlinear least‐squares method with sigmaplot12 (Systat Software Inc., San Jose, CA, USA).

## Results

### Isolation of UDT candidates

All reported plant‐derived PT genes for phenolic substrates belong to the UbiA superfamily (Winkelblech *et al*., 2015), with the PTs responsible for plant‐specialized metabolism showing moderate amino acid identities (30–50%) with another group of UbiA PTs involved in plant primary metabolism (Karamat *et al*., 2014; Wang *et al*., 2014). To search for aromatic PTs in fig, we performed a homology‐based *in silico* screening using primary metabolite‐related UbiA members in *Arabidopsis thaliana* as queries (AtVTE2‐1, AtVTE2‐2, AtPPT1, AtABC4, AtATG4 and AtCOX10, which participate in the biosynthesis of tocopherol, plastoquinone, ubiquinone, phylloquinone, Chl, and haem *a*, respectively) (Table S2a) (Winkelblech *et al*., 2015). A tblastn search performed on an RNA‐seq library prepared from fig fruit latexes yielded three candidate genes, tentatively named UDT*‐*candidates1–3 (Fig. S3a).

UDT‐candidate1 contains a partial PT sequence lacking the 5′‐terminal region; however, it was complemented based on a homologous contig (Fr2001904) identified in an RNA‐seq of *F. religiosa*, another *Ficus* species accumulating FCs (Singh *et al*., 2011), in the OneKP database (Fig. S4). Another contig (Fr2007013) identified in the *F. religiosa* RNA‐seq allowed the extension of the 3′‐UTR sequence of UDT‐candidate1 (Fig. S4). Using the combined sequence information from these three contigs (Fig. S4), two full CDSs were isolated from fig mRNA by RT‐PCR‐based cloning, and these CDSs were named *FcPT1a* and *b*. UDT‐candidates2 and 3 were found to encode identical PT genes harbouring a single silent mismatch in their CDSs and to have highly homologous UTR sequences (Fig. S3). Based on the sequence of UDT‐candidate2, which was longer than that of UDT‐candidate3, two additional CDSs were cloned by the same RT‐PCR approach and named *FcPT2a* and *b*.

The nucleotide sequence identities of the two variants of *FcPT1* and of the two variants of *FcPT2*, including their UTR regions, were > 98%, with each pair of variants being mapped to the same site in a fig draft genome by blastn (Mori *et al*., 2017). *FcPT1a* and *b* correspond to nucleotides 9576–12 597 and 9576–12 602, respectively, in a fig genome scaffold (accession ID: BDEM01000717.1). Both *FcPT2a* and *b* correspond to nucleotides 198 283–200 428 in another scaffold (accession ID: BDEM01000270.1). These findings indicate that each pair of variants represents allelic pairs.

### Polypeptide structures of FcPTs

*Ficus carica* prenyltransferase 1 and 2 polypeptides share 51% amino acid identity, regardless of their variants. tmhmm analysis predicted that the four polypeptides have multiple transmembrane alpha‐helices and chlorop predicted that the N‐terminal regions of these polypeptides have a transit peptide (Fig. S5a). Both pairs of polypeptide sequences possessed the typical structural characteristics of plant aromatic PTs, including Apiaceae UDTs (Winkelblech *et al*., 2015). Two aspartate‐rich motifs were observed in FcPT1a and b, whereas FcPT2a and b have an atypical substitution of a glycine for a glutamine in the first motif (Fig. S5b). Because the substitution was also observed in their contigs (UDT‐candidates2 and 3; Fig. S3a), they probably represent a natural variation. A similar substitution in this conserved sequence was observed in *Rhododendron dauricum PT1*; its native gene possesses an alanine at the same position, with replacement of this alanine by a glutamine reducing catalytic activity (Saeki *et al*., 2018). The four proteins were therefore biochemically characterized.

### Characterization of the U6DT activity of FcPT1

**Figure 2:**
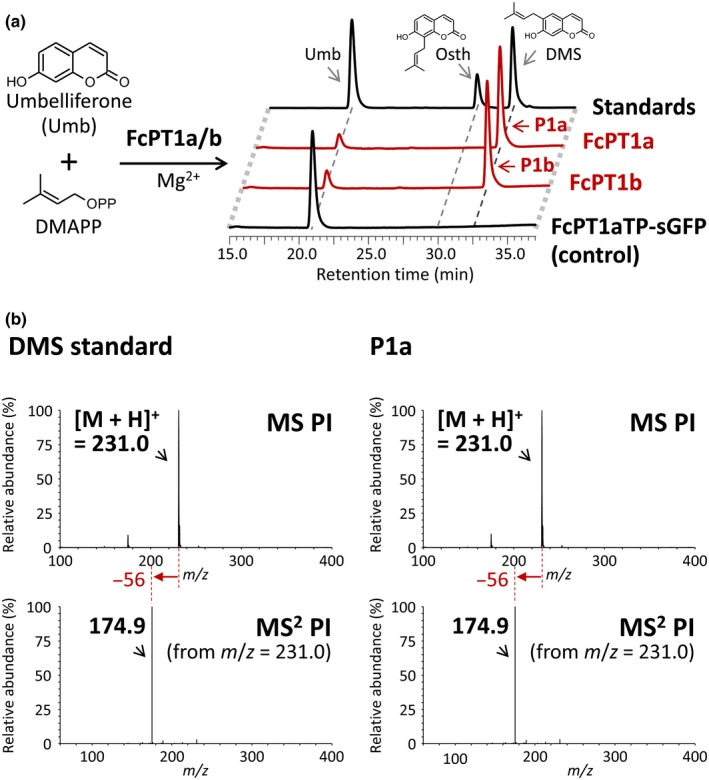
Liquid chromatography‐MS analysis of the umbelliferone dimethylallyltransferase (UDT) reaction mixture of *Ficus carica* prenyltransferase 1 (FcPT1). (a) Ultraviolet chromatograms of UDT reaction mixtures of FcPT1a/b. Chromatograms for FcPT1a, FcPT1b and FcPT1aTP‐sGFP as a negative control are shown at 330 nm on a comparable scale. Microsomes containing *c.* 0.3 µg of total proteins were incubated with 50 µM umbelliferone and 200 µM dimethylallyl diphosphate (DMAPP) in the presence of 10 mM MgCl_2_. (b) Tandem mass spectrometry (MS^2^) spectra of enzymatic reaction products of FcPT1a in the positive ion mode. The loss of 56 mass units is a result of a specific fragmentation to dimethylallyl moieties attached to aromatic rings via C‐C bonds (Simons *et al*., 2009).

To characterize the enzymatic function of FcPTs, their full CDSs were transiently expressed in *N. benthamiana*, and the microsomal fractions prepared from their leaves were used as crude enzymes for *in vitro* assays. Our results showed no enzymatic reaction products when FcPT2a/b microsomes were incubated with different substrate combinations, including the pair of umbelliferone and DMAPP, in the presence of Mg^2+^ as a cofactor (Fig. S6). By contrast, HPLC analysis of UDT reaction mixtures composed of microsomes containing recombinant FcPT1a/b proteins yielded an enzymatic reaction product concomitant with the consumption of umbelliferone (Figs 2a, S6). This product was identified as DMS by direct comparison of its retention time and tandem mass spectrometry (MS^2^) spectrum with those of a standard molecule (Fig. 2). Moreover, this product did not appear in any control incubations (Figs 2a, S7a). In contrast to Apiaceae U6DTs, which yielded osthenol as a by‐product (Karamat *et al*., 2014; Munakata *et al*., 2016), FcPT1a/b did not. Because both FcPT1a/b variants yielded the same results, we focused on FcPT1a in subsequent investigations. The apparent *K*_m_ values of FcPT1a for umbelliferone and DMAPP were determined to be 35 ± 4 and 17 ± 1 µM, respectively (Fig. S7b), which is similar to those of parsley PcPT1 (21 ± 3 µM for umbelliferone and 80 ± 10 µM for DMAPP) (Karamat *et al*., 2014).

### Substrate preference of FcPT1

**Figure 3:**
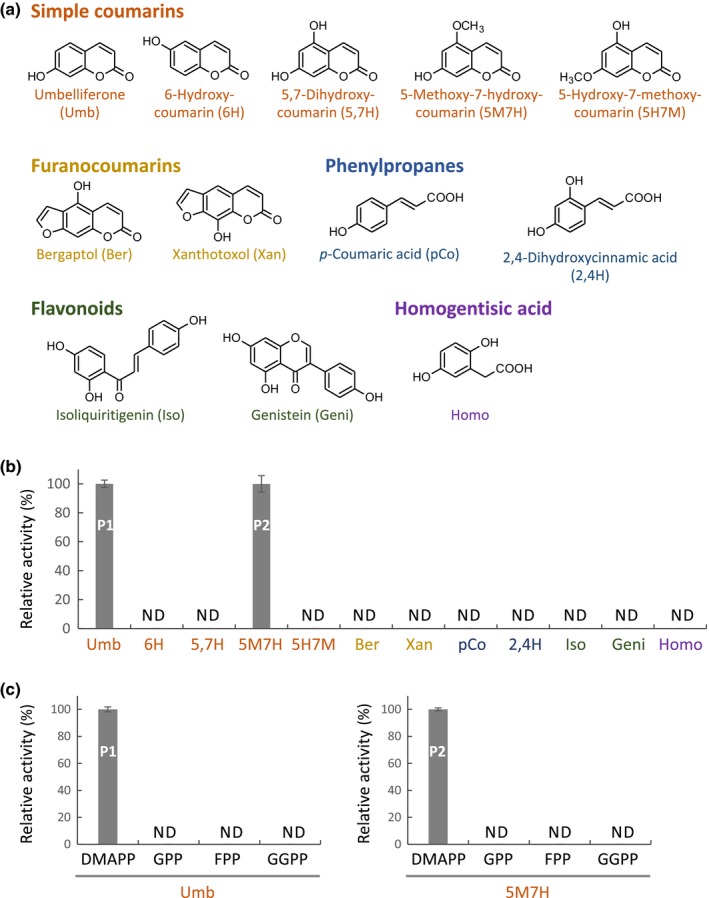
Substrate specificity of *Ficus carica* prenyltransferase 1a (FcPT1a). (a) Aromatic substrates tested. (b) Prenyl acceptor preference in use of dimethylallyl diphosphate (DMAPP) as a prenyl donor. (c) Prenyl donor preference in use of umbelliferone (Umb, left) or 5‐methoxy‐7‐hydroxycoumarin (5M7H, right) as a cosubstrate. In (c), 100 µM of prenyl diphosphates were incubated with the other components. Values are expressed as the means ± SE (*n* = 3 each). Dimethylallylated 5M7H (P2) was quantified as equivalent to 5M7H. ND, not detected. GPP, geranyl diphosphate; FPP, farnesyl diphosphate; GGPP, geranylgeranyl diphosphate.

Substrate specificity of FcPT1a for prenyl acceptors was evaluated with various aromatic compounds using DMAPP as a prenyl donor. Incubations with various simple coumarins and FCs showed that this enzyme recognized umbelliferone and 5‐methoxy‐7‐hydroxycoumarin (5M7H) as prenyl acceptors (Figs 3a,b, S8). All other simple coumarin/FC derivatives tested with different substitution patterns were not transformed (Fig. 3a,b). This clear preference strongly suggests that FcPT1a requires a hydroxyl moiety on C7 of the coumarin structure, although this moiety was not sufficient, as 5,7‐dihydroxycoumarin was not transformed (Fig. 3b). This enzyme did not react with other phenolic compounds, including phenylpropanes, flavonoids and homogentisic acid (Fig. 3a,b).

The specificity of FcPT1a for prenyl donor substrates was also assessed using geranyl diphosphate, farnesyl diphosphate and GGPP in the presence of umbelliferone or 5M7H, but we could not detect any products (Fig. 3c). These *in vitro* experiments indicated that the recombinant FcPT1a specifically transfers a dimethylallyl moiety to umbelliferone and 5M7H. The enzymatic reaction product of 5M7H has not been found in fig plants and is thus presumed to be 6‐dimethylallylated 5M7H (Fig. S8c) based on the 6‐specific prenylation of umbelliferone by this enzyme (Fig. 2).

### Expression profile of FcPT1

**Figure 4:**
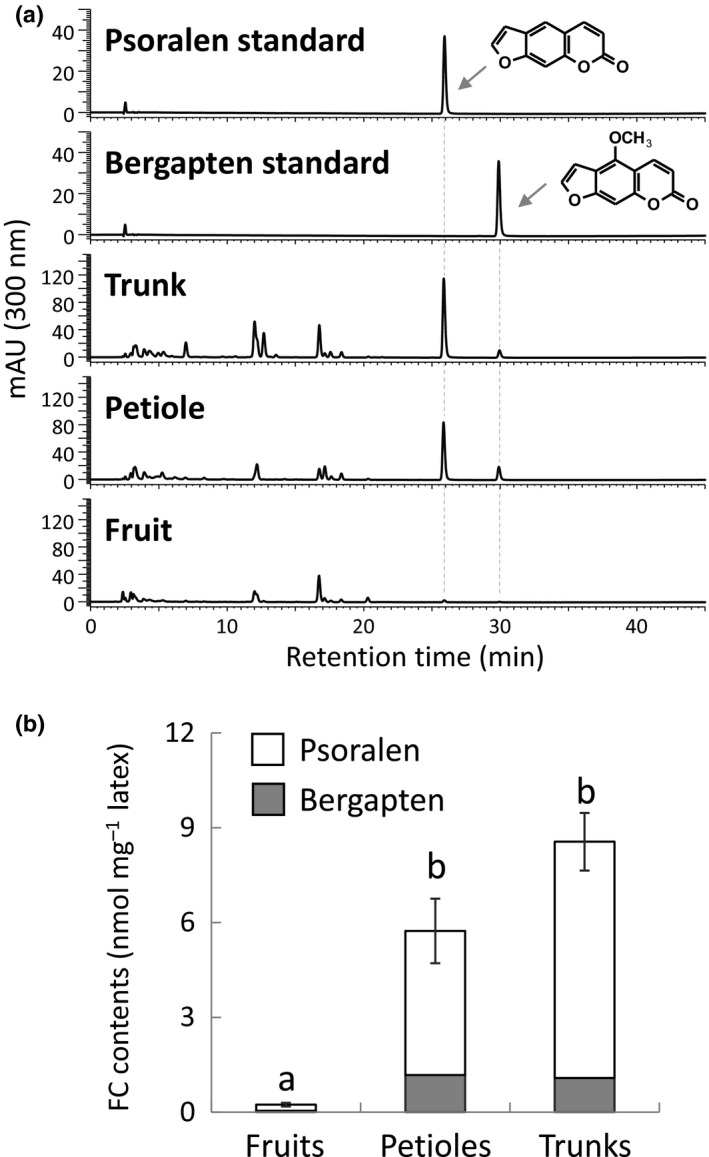
Furanocoumarin (FC) contents in latexes collected from different fig organs. Ultraviolet chromatograms (a) and total FC contents calculated by quantification of psoralen and bergapten (b) of methanol extracts of latexes collected from different fig organs. Values are means ± SE (*n* = 5 each). Letters indicate statistical significance (*P* < 0.05) by the Steel–Dwass test.

**Figure 5:**
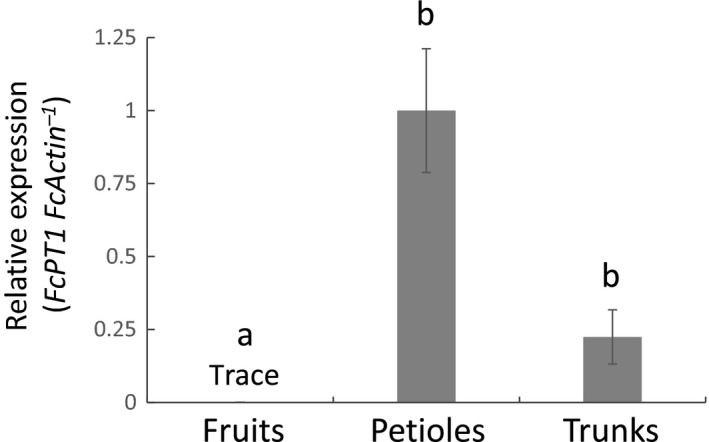
Real‐time PCR for *Ficus carica prenyltransferase 1* (*FcPT1*) expression in latexes from different fig organs. Levels of *FcPT1* expression by fig latex samples from fruits (*n* = 5), petioles (*n* = 4), and trunks (*n* = 5) were normalized relative to levels of *FcActin* expression and expressed relative to the normalized level in petiole latex. Values are means ± SE. Letters indicate statistical significance (*P* < 0.05) by the Steel–Dwass test.

Furanocoumarin contents vary among fig organs (Oliveira *et al*., 2009), but no reports have described the distribution of FC molecules through latexes from fig fruits, petioles and trunks (Fig. S2), from which comparable RNA‐seq datasets were constructed (Kitajima *et al*., 2018). We therefore measured the total FC contents in these three latex preparations by quantification of psoralen and bergapten, considering the large majority of FC derivatives in fig (Oliveira *et al*., 2009). The total FC contents in the petiole and trunk latex preparations were 24‐ and 35‐fold higher, respectively, than those in fig fruit latex (Figs 4, S9), suggesting large variations in FC production by these tissues. Subsequent qRT‐PCR analysis revealed similar relative levels of *FcPT1* expression in these latex preparations (Figs 4, 5), which suggested that this gene is involved in FC biosynthesis.

Because *FcPT1a*/*b* were originally isolated from an RNA‐seq library constructed from fig fruit latexes, we searched for other *UDT* candidates in comparable RNA‐seq libraries (Kitajima *et al*., 2018). A tblastn search using FcPT1a/b and six *Arabidopsis* UbiA PTs involved in primary metabolism identified 69 contigs that could be classified in the UbiA superfamily (Table S3). These 69 contigs were found to cluster in three groups. The first group included 19 contigs, the gene products of which showed the highest amino acid identities with FcPT1a/b among the eight earlier‐described queries. The second group was composed of a single contig showing the highest amino acid identity with AtPPT1 at a moderate level below the threshold set at 58%, which corresponds to the amino acid identity between AtPPT1 and its orthologue in *Oryza sativa*, OsPPT1. These two groups of 20 contigs were considered as *UDT* candidates (Table S3a). The third group included the remaining 49 contigs and were annotated as orthologues of the primary PTs because they showed amino acid identities with one of the queries over the thresholds, set at 63%, 66%, 53%, 76%, and 55% for VTE2‐1, VTE2‐2, ABC4, ATG4, and COX10, respectively, as described for PPT (Table S3b).

**Figure 6:**
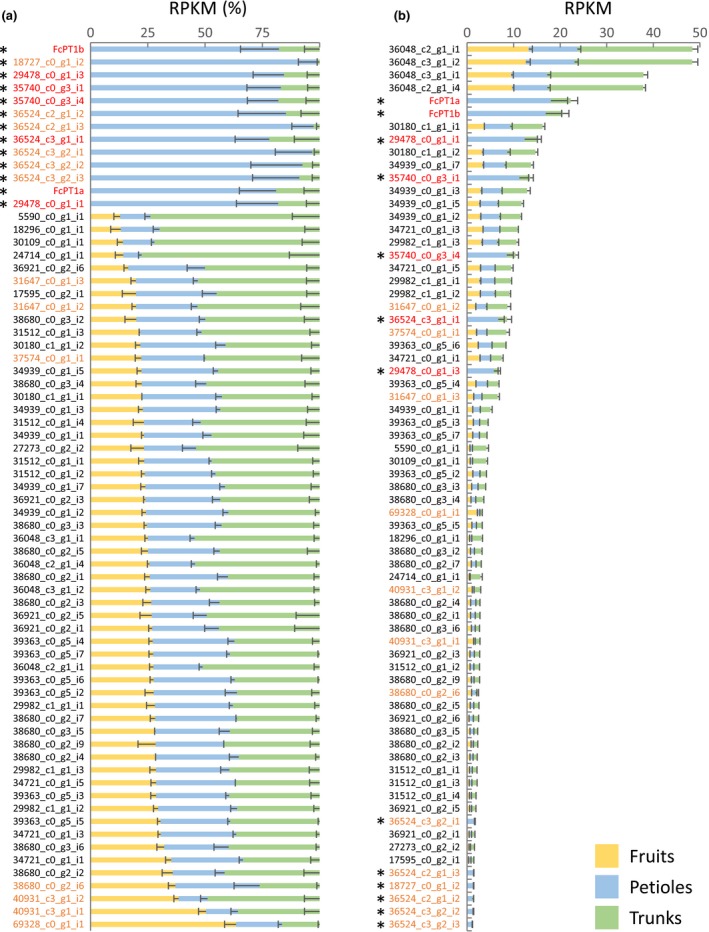
Reads per kilobase of exon model per million mapped reads (RPKM)‐based organ‐specific abundance of contigs assigned to the UbiA superfamily. Contigs assigned to the UbiA superfamily were listed according to RPKM ratios for fruit latex (RPKM for fruit latex/ total RPKM for the three latexes) (a) and total RPKM (b). Asterisks indicate low ratios for fruit latex. Contigs predicted to possess unknown functions or umbelliferone 6‐dimethylallyltransferase (U6DT) in Supporting information Table S3 are highlighted in orange or red letters, respectively. RPKMs (means ± SE; *n* = 3 each) are shown (Kitajima *et al*., 2018). Three contigs (31647_c0_g1_i2, 31647_c0_g1_i3 and 37574_c0_gi_i1) correspond to *Ficus carica prenyltransferase 2a/b.*

The reads per kilobase of exon model per million mapped reads (RPKM) analysis of the fruit latex indicated that 13 of the 69 contigs (asterisks in Fig. 6a; Table S3a) had low ratios and may participate in the production of FCs (Fig. 4). All were annotated as U6DT or unknown functions (Table S3a) and belonged to group 1. These 13 contigs could be split into two subgroups with high and low RPKM values (Fig. 6b). The high RPKM subgroup contained seven contigs annotated as U6DT, which encode *FcPT1a/b*, partial CDSs almost identical (> 99%) to *FcPT1a/b*, and partial CDSs of *FcPT1a/b* with yet‐to‐be spliced introns (highlighted in red in Fig. 6; Table S3a)*.* These *in silico* analyses provided further evidence that *FcPT1a/b* are the most promising candidates for *UDT*. The remaining six contigs with low RPKM values were mapped to two close genomic loci (accession ID: BDEM01000105.1), one of which contains a full gene structure. However, its gene product encoded by 36524_c3_g2_i2 is rather divergent (< 55% identity) from FcPT1 or 2, which is the similar divergence from Moraceae PTs for other phenolic groups, that is, *Morus alba* isoliquiritigenin dimethylallyltransferase (MaIDT) and *Cudrania tricuspidata* IDT (CtIDT), both specific to flavonoids, and *M. alba* oxyresveratrol geranyltransferase (MaOGT), specific to stilbenoids (Wang *et al*., 2014; Zhong *et al*., 2018), suggesting its function is different from that of UDT. Three other contigs, 31647_c0_g1_i2, 31647_c0_g1_i3, and 37574_c0_g1_i1, showed relatively low expression ratios for the fruit latex and total expression levels comparable to *FcPT1‐*related contigs (Fig. 6), but they were all almost identical (> 99%) to *FcPT2a/b*.

### Subcellular localization of FcPT1

**Figure 7:**
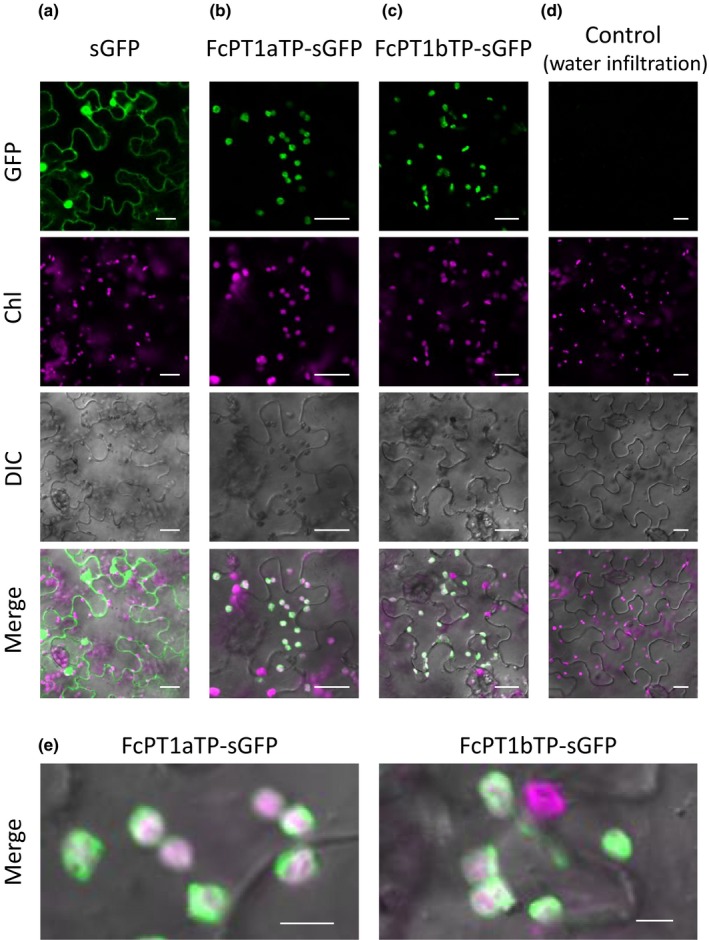
Microscopic observation of *Ficus carica* prenyltransferase 1 transit peptide (FcPT1TP)‐synthetic green fluorescent protein (sGFP) expressed in *Nicotiana benthamiana* epidermal cells. (a–c) sGFP (a), FcPT1aTP‐sGFP (b), and FcPT1bTP‐sGFP (c) were transiently expressed in *N. benthamiana* leaves by agroinfiltration. Water infiltrated into the leaves was the negative control (d). Rows from the top indicate images of GFP signalling, Chl autofluorescence, differential interference contrast (DIC) images, and merged images. Enlarged images (e) are also shown for FcPT1aTP‐sGFP (b) and FcPT1b‐sGFP (c). Bars; 20 µm (a–d); 5 µm (e).

The subcellular localization of FcPT1 *in planta* was assessed using both sGFP‐chimeric proteins harbouring the first 72 and 70 amino acids of FcPT1a (FcPT1aTP‐sGFP) and FcPT1b (FcPT1bTP‐sGFP), respectively, as the N‐terminal regions of the FcPT1 variants show uneven sequence divergence (Fig. S5b). The proteins were transiently expressed in *N. benthamiana* leaves by agroinfiltration, and GFP fluorescence was monitored by confocal microscopy. The GFP signal for both chimeric proteins localized to chloroplasts (Fig. 7). These results suggest that FcPT1a/b localize to plastids, which is consistent with the synthesis of prenyl donors for UbiA PTs, including DMAPP, via the MEP pathway (Akashi *et al*., 2009; Saeki *et al*., 2018).

### Phylogenetic analysis of FC biosynthetic enzymes

**Figure 8:**
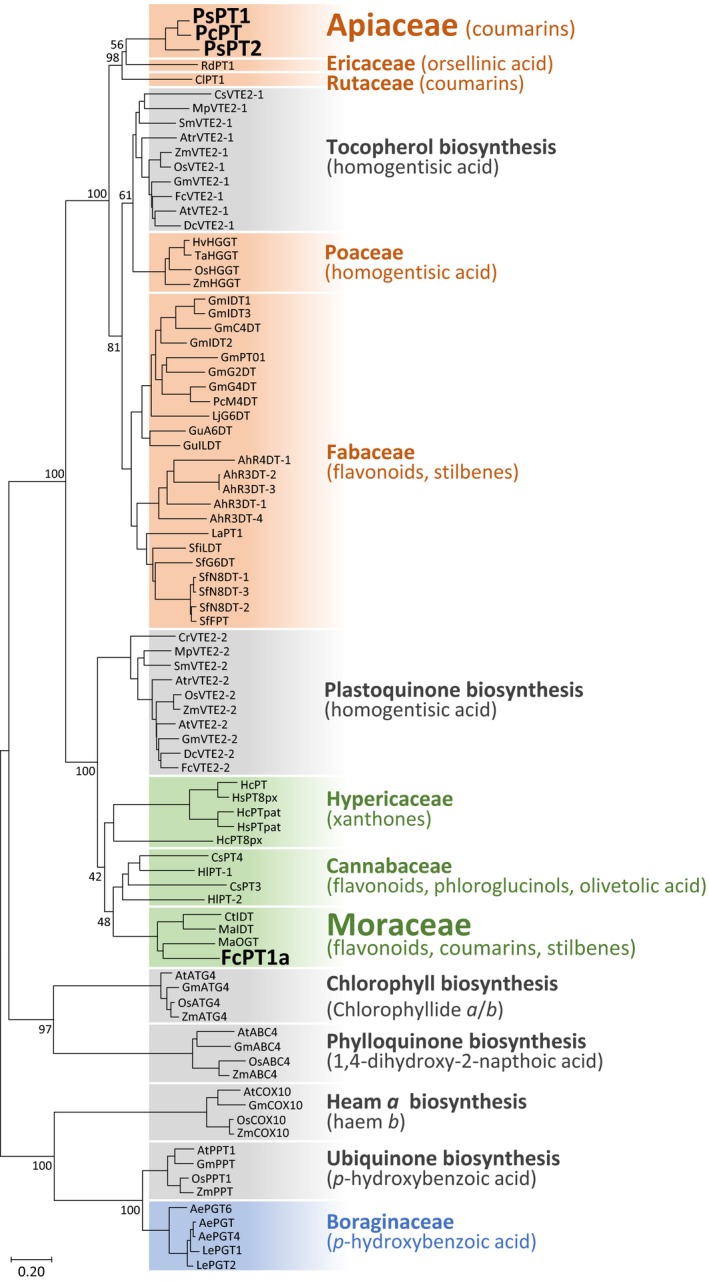
Phylogenetic relationship between *Ficus carica* prenyltransferase 1a (FcPT1a) and Apiaceae umbelliferone dimethylallyltransferases (UDTs) in the UbiA superfamily. A neighbour‐joining phylogenetic tree of UbiA prenyltransferase (PT) polypeptides was constructed. The results of 1000 bootstrap tests (maximum 100) are shown for nodes generating clades and more upstream nodes. Clades of primary and specialized metabolic pathways are coloured grey, and others depend on their estimated ancestor enzymes (orange, VTE2‐1 origin; green, VTE2‐2 origin; blue, PPT origin). Their aromatic substrates are shown in parentheses, and UDTs are highlighted in bold and larger font. The bar represents an amino acid substitution rate per site of 0.2. Abbreviations for plant names and enzyme names are given together with their accession numbers in Supporting Information Table S2.

A phylogenetic tree was constructed using UbiA PT polypeptides, including FcPT1a and Apiaceae UDTs (PcPT1, PsPT1 and PsPT2). In the tree, primary metabolite‐related PTs are grouped by their physiological/biochemical functions, with PTs derived from different plant species being grouped into one clade. By contrast, specialized metabolite‐related UbiA PTs generated the other clades close to the VTE2‐1, VTE2‐2 or PPT clade. These findings appear to reflect an ancestral gene function (Fig. 8). In this analysis, three Apiaceae UDTs are close to the VTE2‐1 clade, whereas FcPT1a is included in a VTE2‐2‐related clade. Interestingly, this second cluster gathered other specialized Moraceae PTs regardless of their different enzymatic functions. This Moraceae clade is also next to the clade of specialized PTs from Cannabaceae (Tsurumaru *et al*., 2012; Li *et al*., 2015) (Fig. 8). Both Cannabaceae and Moraceae are classified as Rosales (Chase *et al*., 2016).

**Figure 9:**
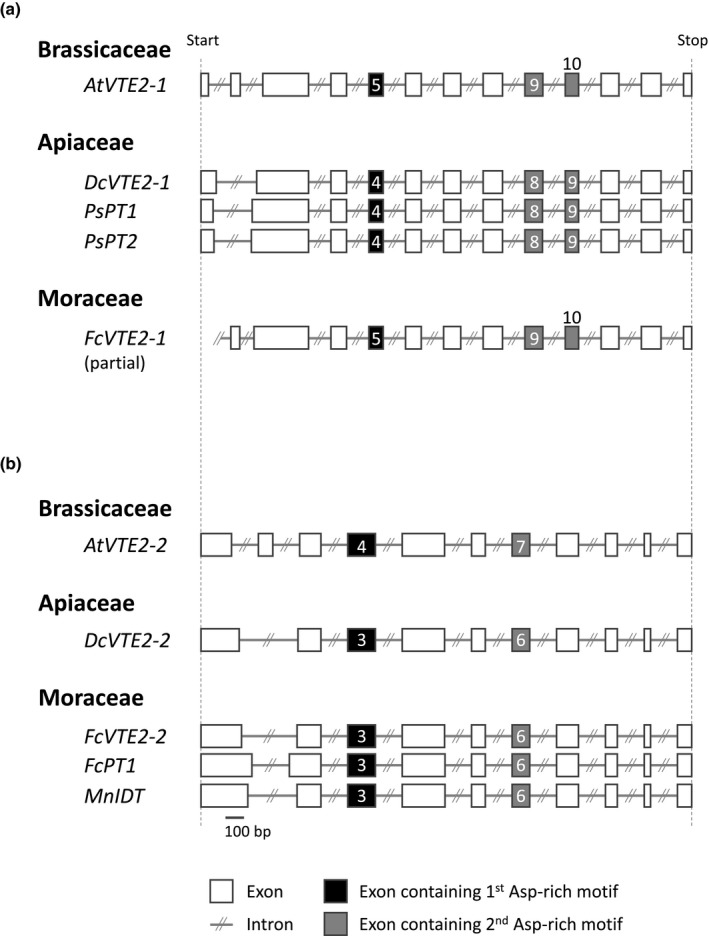
Exon organization of *umbelliferone dimethylallyltransferases* (*UDTs*) and their relative prenyltransferase (PT) genes. (a, b) Exon‐intron structures of Apiaceae *UDTs* (a) and *Ficus carica PT1* (*FcPT1*) (b) along with related *PT* members. Exons containing the first and second aspartate‐rich motifs are numbered and shown in black and grey, respectively. Bar, 100 bp. The genomic sequences of *Daucus carota VTE2‐1* (*DcVTE2‐1*) and *FcVTE2‐1* and *FcVTE2‐2* were searched by tblastn analysis of the whole‐genome shotgun contigs in NCBI using *Arabidopsis thaliana* VTE2‐1 (AtVTE2‐1) and AtVTE2‐2 as queries, respectively. The genomic sequence of *Morus notabilis isoliquiritigenin DT* (*MnIDT*), which share 98% identity with *Morus alba IDT* (*MaIDT*) in coding sequence, was found in MorusDB. Detailed information for PT genes is shown in Supporting Information Table S4. The exons of *FcVTE2‐1* are numbered in reference to those of *AtVTE2‐1.*

Phylogenetic analysis was completed by comparing the genomic sequences of Moraceae and Apiaceae *UDTs*. We first compared the gene sequences of VTE2‐1s and VTE2‐2s in a broad taxonomical range from chlorophytes to angiosperms, including species from these two families (fig and *Daucus carota* (carrot)) (Fig. 8). Among these genes, *VTE2‐1/2‐2* from *A. thaliana* and *VTE2‐2* from *Chlamydomonas reinhardtii* were already functionally characterized (Sadre *et al*., 2006). Except for the 5′‐terminal regions of these genes containing divergent transit peptides, exon structures of both *VTE2‐1s* and *VTE2‐2s* were highly conserved over angiosperms (Figs 9, S10a,b). However, the total number and length of exons clearly differ between the conserved structures of the two *PT* groups (Figs 9, S10a,b), which is exemplified by the difference in the position of the two aspartate‐rich motifs. The lengths of introns in each gene are not well conserved in either PT group (Fig. S10c,d).

The exon structures of *VTE2‐1s* and *VTE2‐2s* were compared with those of *PsPT1*/*2* (Munakata *et al*., 2016) and *FcPT1a*. This comparison revealed that Apiaceae *UDTs* and *FcPT1* possess conserved exon structures of *VTE2‐1s* and *VTE2‐2s*, respectively (Figs 9, S10). These phylogenetic tree and exon structures strongly suggest that Apiaceae and Moraceae plants recruited *UDT* from different ancestral genes, that is, *VTE2‐1* and *VTE2‐2*, respectively.

A broader view of the FC pathway was provided by *in silico* analysis of genes encoding enzymes responsible for the formation of umbelliferone, upstream of the prenylation step (Fig. 1). In angiosperms, including FC‐producing species in Apiaceae and Rutaceae, this reaction is performed by a *p*‐coumaroyl CoA 2′‐hydroxylase (C2′H) belonging to the DOXC30 group in the 2‐oxoglutarate‐dependent dioxygenase superfamily (Roselli *et al*., 2017; Vialart *et al*., 2012; Kawai *et al*., 2014). Phylogenetic comparisons of putative fig C2′H proteins previously screened in the latex RNA‐seq libraries (Kawai *et al*., 2014; Kitajima *et al*., 2018) showed the clustering of them in the DOXC30 clade (Fig. S11). In contrast to the UDT step, it is thus possible that the C2′H reaction in Moraceae is catalysed by enzymes orthologous to DOXC30s in other angiosperms.

Recently, a new gene encoding an enzyme catalysing *trans‐cis* isomerization and lactonization of *o*‐hydroxycinnamoyl‐CoA (Vanholme *et al*., 2019) was reported, which serves as an alternative route to the nonenzymatic process in coumarin skeleton formation (Fig. 1). This *A. thaliana COUMARIN SYNTHASE* (*COSY*) is responsible for the formation of scopoletin and esculetin (Vanholme *et al*., 2019), neither of which is demonstrated to be incorporated into FCs in plants. However, this enzyme might contribute to umbelliferone synthesis in other species. An *in vitro* experiment demonstrated that AtCOSY is able to synthesize this FC precursor from 2,4‐dihydroxycinamoyl‐CoA (Vanholme *et al*., 2019). The homologous genes of AtCOSY are conserved in various angiosperm taxa, including FC‐rich species, that is, fig, *Angelica archangelica* (Apiaceae), *Bituminaria bituminosa* (Fabaceae) and *Citrus* × *paradisi* (Rutaceae) (Murray *et al*., 1982) (Fig. S12). Future studies should include functional characterization of these dioxygenases and COSY enzymes.

## Discussion

This study identified *FcPT1*, a *U6DT* involved in FC biosynthesis in fig latexes. This enzyme belongs to the UbiA superfamily and possesses characteristics typical of PT members accepting phenolic substrates, that is, multiple transmembrane regions, two aspartate‐rich motifs, and an *N*‐terminal transit peptide (Winkelblech *et al*., 2015). Enzymatic characterization showed that FcPT1 has narrow substrate preferences for prenyl donors and acceptors, similar to Apiaceae UDTs (Karamat *et al*., 2014; Munakata *et al*., 2016). These findings confirmed that this U6DT is responsible for the enzymatic transformation of umbelliferone to DMS in figs. Interestingly, FcPT1 can prenylate 5M7H as much as umbelliferone *in vitro*, supporting the previously advanced hypothesis that another route leads to the production of FCs. In the fig FC pathway, hydroxylation at the C5 position followed by *O*‐methylation, not only for psoralen but also for marmesin, may lead to a 5‐*O*‐methoxy moiety of bergapten (Murray *et al*., 1982), suggesting a grid‐type biosynthetic pathway in this plant. Our biochemical data suggest that the transformation of umbelliferone into 5M7H before prenylation could be an alternative route for the formation of bergapten in fig. However, this metabolic route may be somewhat minor, as tracer experiments showed that 5M7H is less efficiently incorporated into bergapten than umbelliferone (Marciani *et al*., 1974).

Unlike similarities of both polypeptide sequence and enzymatic properties of UDTs in fig and Apiaceae species, our phylogenetic analysis together with the comparison of gene structures strongly suggests that *FcPT1* and Apiaceae *UDTs* evolved from different ancestors. As FCs were isolated from phylogenetically distant plants, two alternative assumptions concerning the emergence of the FC pathway were suggested: either it appeared in a common ancestor and then disappeared during evolution, or it appeared independently in the different taxa. If referring to the first hypothesis, this possibility would mean that both *VTE2‐1*‐ and *VTE2‐2*‐related *UDTs* were present in a common ancestor followed by disappearance of a gene during evolution. This hypothesis sounds unlikely, as plant species harbouring a set of secondary metabolic UbiA PTs related to multiple primary metabolic UbiA PTs (e.g. from both VTE2‐1 and VTE2‐2) have not been reported to date (Li *et al*., 2015; Munakata *et al*., 2016; Yoneyama *et al*., 2016). Our phylogenetic analysis also showed that despite diverse enzymatic functions, all of the reported UbiA PTs involved in secondary metabolism from Rosales (including Moraceae and Cannabaceae) are clustered close to the VTE2‐2 clade, whereas those from Fabales are close to the VTE2‐1 clade (Wang *et al*., 2014; Zhong *et al*., 2018). Rosales and Fabales are taxonomic neighbours (Fig. S1), suggesting that gene duplication and neofunctionalization events of *VTE2‐2* and *VTE2‐1* after the divergence between these two taxa have led to taxon‐specific metabolic pathways in Rosales and Fabales, respectively. Therefore, *UDTs* were probably independently acquired between Moraceae and Apiaceae in a convergent evolutionary manner, supporting the independent acquisition of the FC pathway between the two families, as stated by the second hypothesis. This evolutionary trajectory is in line with previous reports describing the convergent evolution of flavonoid and stilbene PTs in Moraceae and Fabaceae (Wang *et al*., 2014; Zhong *et al*., 2018) and suggests that Moraceae developed the linear FC pathway independently of Fabaceae and probably also from Rutaceae, the other major FC‐producing families (Murray *et al*., 1982).

In addition to the four major FC‐producing taxa, FCs were found in 11 families classified into seven plant orders (Murray *et al*., 1982). Owing to the development of analytical tools, FCs have been isolated from other angiosperms, such as *Dioscorea communis* (Discoreales) (Zerargui *et al*., 2015), as well as from other plants outside angiosperms, such as *Pseudolarix kaempferi* (Pinales, Gymnosperms) (Cai *et al*., 2012) and *Selaginella moellendorffii* (Selaginellales) (Weng & Noel, 2013) in the last decade. Future progress in FC research may find that the FC pathway is widely distributed throughout the plant kingdom by convergent evolutionary processes.

Several hypotheses may explain the independent appearance of the pathway in various plant taxa. The first hypothesis is related to the toxicity of FCs in a broad range of organisms. Under UV‐A irradiation, linear FC molecules intercalate into double‐stranded DNAs by covalent cross‐linking to pyrimidine bases, potentially inhibiting DNA replication and transcription (Kitamura *et al*., 2005; Bourgaud *et al*., 2006). Linear FCs can also inactivate several P450 enzymes, one of the most ubiquitous enzyme families among organisms (Lin *et al*., 2012; Gravot *et al*., 2004). These toxic activities were reported to be effective against bacteria, fungi, plants, humans and even DNA viruses (Murray *et al*., 1982). Thus, in response to stresses, unrelated plant taxa may have independently developed a linear FC pathway. In fig trees, FCs were more concentrated in latexes of trunk and petioles than those of fruits, with FC contents also being reported to be considerably higher in leaves than in fruits (Oliveira *et al*., 2009). By contrast, FC contents are higher in young fruits than in other organs of *R. graveolens* (Milesi *et al*., 2001), suggesting that independently evolved FCs may differ in their distribution among plant tissues. This difference may be related to differences in plant defence strategy among unrelated taxonomical groups. Furthermore, FC production in Apiaceae species is induced by both insect herbivores and fungal infection, suggesting that plants have taken advantage of the toxicities of FCs (Zangerl, 1990; Schmelzer *et al*., 1989).

The second hypothesis that may explain the convergence of this pathway is associated with the small number of biosynthetic reactions required to produce these toxic molecules. Our *in silico* analyses of the DOXC30 subfamily and COSY homologues suggest that the Moraceae and Apiaceae lineages inherited umbelliferone synthase from their common ancestor. Because psoralen is sufficient to cause both genotoxicity and mechanism‐based inhibition of P450 enzymes (Kitamura *et al*., 2005; Gravot *et al*., 2004), plants required only three enzymes, U6DT, MS, and PS, to produce this efficient defence molecule (Murray *et al*., 1982). The simplicity of this pathway may have led to its appearance in different taxa. In comparison, complex specialized metabolic pathways requiring more than a dozen biosynthetic steps, such as those involving the biosyntheses of paclitaxel and vinblastine, are more likely to be monophyletic in plants (Croteau *et al*., 2006; Caputi *et al*., 2018).

The ability of several unrelated plant species to independently produce a particular metabolite has been reported for various groups of metabolites, including alkaloids and terpenes (Pichersky & Lewinsohn, 2011). The identity, or parallelism, of the processes involved in convergent evolution varies on a case‐by‐case basis. One example of low parallelism is the synthesis of aminobenzoic acid in corn (Poaceae) and *Vitis labrusca* (Vitaceae). Both plants produce methyl anthranilate by a single reaction but start with different substrates and utilize distinct enzyme families (Pichersky & Lewinsohn, 2011). By contrast, an example of high parallelism is the synthesis of caffeine in coffee and tea, which requires multiple methylation reactions but differs only slightly between these species. Xanthosine, a common precursor, is transformed through three consecutive *N*‐methyltransferase (*N‐*MT) reactions, which are biochemically similar in both plants (Huang *et al*., 2016). The *N*‐MT reactions in coffee are catalysed by three different enzymes specifically dedicated to individual steps, whereas only two *N*‐MT enzymes are required in tea (Huang *et al*., 2016). A phylogenetic analysis revealed a clear sequence divergence between the enzymes isolated from these plants (Huang *et al*., 2016). Other plants that have independently evolved a caffeine production pathway include cacao, guarana and orange. In all of these plant taxa, the enzymes recruited for the synthesis of caffeine are *N*‐MTs belonging to the SABATH superfamily (Huang *et al*., 2016).

The biosynthesis of FCs probably includes a similar high parallelism. In contrast to the caffeine pathway, however, the FC pathway is more complicated at the molecular level as a result of the involvement of unrelated enzyme families, such as the UbiA, P450 and SABATH superfamilies (Hehmann *et al*., 2004; Larbat *et al*., 2007; Karamat *et al*., 2014). Similar high genetic complexity is observed in the convergent evolution of the biosynthetic pathways of pyrrolizidine alkaloids (Ober & Kaltenegger, 2009) and benzoxazinoids (Dick *et al*., 2012). The genetic simplicity or complexity of a pathway may be associated with its rapidity of appearance during plant evolution. For example, Huang and collaborators used a computational approach to resurrect the ancestral *N*‐MT sequence located at the phylogenetic branching point between two *N*‐MTs specifically involved in the caffeine pathway of orange. This ancestral enzyme could be neofunctionalized to become almost equivalent to the present two *N*‐MTs by different single mutations (Huang *et al*., 2016). Such a reconstruction approach may enable us to assume the rapidity of construction of the FC pathway in a plant taxon by the mutations necessary for neofunctionalization of reconstructed ancestors towards FC‐specialized enzymes. The rapidity, together with genetic simplicity and a minimization of the number of involved enzymes, can facilitate the independent emergence of pathways involved in the biosynthesis of the same molecules in different plant species.

Along with constructing biosynthetic pathways, plants must frequently develop mechanisms of resistance to their own active compounds. This strategy may be similar to those developed for sequestering the cytosol and nucleus, which are important for plant acquisition of energy and reproduction. Apiaceae and Rutaceae species export a large quantity of FC molecules into hydrophobic extracellular compartments, called oil ducts and oil cavities, respectively (Reinold & Hahlbrock, 1997; Voo *et al*., 2012). The strategy differs significantly from those of fig latexes, which are living cells producing high amounts of FCs (9 ± 2 mM in fig trunk latexes, with 1 µl of fig latex weighing 1 mg), roughly comparable to those in oil cavities in Rutaceae (24–30 mM in grapefruit oil cavities) (Voo *et al*., 2012). The high intracellular accumulation of FCs suggests that currently unrevealed fig‐specific mechanisms circumvent the self‐toxicity of endogenous FC molecules.

In conclusion, our phylogenetic and gene structure analyses support the convergent evolution of FCs in plants by comparing UDTs in Moraceae and Apiaceae. Similar evolutionary strategies may be employed in other plant taxa, ranging from angiosperms to *Selaginellales*. Further investigations into FC metabolism in different unrelated plant taxa are necessary to provide more comprehensive insights into the convergence of plant‐specialized metabolic pathways, as well as into divergent and convergent strategies developed by plants to coexist with the self‐toxicities of these convergently acquired metabolites.

## Author contributions

RM, AS, and KY designed the research. SK maintained the fig trees and constructed the RNA‐seq library from fig fruit latexes. RM and SK performed *in silico* screening of the RNA‐seq libraries for *UDT* and *C2′H* candidates. GG, SV, HB, FB and AH sequenced BAC clones of the parsnip genome. RM quantified FC derivatives in different fig latex types, isolated cDNAs encoding *FcPTs*, performed qRT‐PCR analysis of *FcPT1a/b*, constructed the phylogenetic trees of UbiA PTs and DOXCs, and analysed gene structures of UbiA *PT* genes. RM, TT, KT, and TI analysed the subcellular locations of FcPT1a/bTP‐sGFP. RM and AN biochemically characterized FcPT1a/b. JG maintained HPLC, UPLC‐MS and LC‐LTQ‐Linear Ion Trap‐MS and optimized their conditions for this research. RM, SK AH, and KY interpreted the results, and RM, AH, and KY wrote the manuscript.

## Supporting information

Please note: Wiley Blackwell are not responsible for the content or functionality of any Supporting Information supplied by the authors. Any queries (other than missing material) should be directed to the *New Phytologist* Central Office.

**Fig. S1** Distribution of FCs in angiosperms.
**Fig. S2** Latexes from fig organs.
**Fig. S3** Contigs of an RNA‐seq prepared from fig fruit latexes.
**Fig. S4** *In silico* construction of a putative UDT cDNA for isolation of *FcPT1*.
**Fig. S5** *In silico* feature analysis of FcPT polypeptides.
**Fig. S6** *In vitro* assay of FcPT2.
**Fig. S7** Enzymatic properties of FcPT1a.
**Fig. S8** 5M7H:dimethylallyltransferase (DT) activity of FcPT1.
**Fig. S9** UV spectra of FC compounds in fig latexes.
**Fig. S10** Gene structures of UDTs and their relatives.
**Fig. S11** *In silico* analysis of fig C2′H candidates.
**Fig. S12** *In silico* analysis of angiosperm COSY homologues.
**Table S1** Primer list.
**Table S2** PT polypeptides used for *in silico* analyses.
**Table S3** Contigs belonging to the UbiA superfamily in the comparable RNA‐seq libraries among different latex types.
**Table S4** PT genes used for gene structure analysis.

## Data Availability

Nucleotide sequences coding for FcPT1a (LC369744), FcPT1b (LC369745), FcPT2a (LC369746) and FcPT2b (LC369747) and the genomic sequences of *PsPT1* (MK205179) and *PsPT2* (MK205180) are available in the NCBI database.
