# Supplementary material for: Convergent evolution of the UbiA prenyltransferase family underlies the independent acquisition of furanocoumarins in plants
Source: New Phytol. 2019 Nov 19;225(5):2166–82. doi: 10.1111/nph.16277 (PMC7028039; doi:10.1111/nph.16277)
Supplement: Supplementary file 1 — Fig. S1 Distribution of FCs in angiosperms. Fig. S2 Latexes from fig organs. Fig. S3 Contigs of an RNA‐seq prepared from fig fruit latexes. Fig. S4 In silico construction of a putative UDT cDNA for isolation of FcPT1. Fig. S5 In silico feature analysis of FcPT polypeptides. Fig. S6 In vitro assay of FcPT2. Fig. S7 Enzymatic properties of FcPT1a. Fig. S8 5M7H:dimethylallyltransferase (DT) activity of FcPT1. Fig. S9 UV spectra of FC compounds in fig latexes. Fig. S10 Gene structures of UDTs and their relatives. Fig. S11 In silico analysis of fig C2′H candidates. Fig. S12 In silico analysis of angiosperm COSY homologues. Table S1 Primer list. Table S2 PT polypeptides used for in silico analyses. Table S3 Contigs belonging to the UbiA superfamily in the comparable RNA‐seq libraries among different latex types. Table S4 PT genes used for gene structure analysis. [file NPH-225-2166-s001.pdf]

New Phytologist

New Phytologist Supporting Information

Article title:

Convergent evolution of the UbiA prenyltransferase family underlies the independent acquisition of furanocoumarins in plants

Authors:

Ryosuke Munakata, Sakihito Kitajima, Andréïna Nuttens, Kanade Tatsumi, Tomoya Takemura, Takuji Ichino, Gianni Galati, Sonia Vautrin, Hélène Bergès, Jérémy Grosjean, Frédéric Bourgaud, Akifumi Sugiyama, Alain Hehn, and Kazufumi Yazaki

Article acceptance date: 9 October 2019

The following Supporting Information is available for this article:

Fig. S1 Distribution of FCs in angiosperms

Fig. S2 Latexes from fig organs

Fig. S3 Contigs of an RNA-seq prepared from fig fruit latexes

Fig. S4 *In silico* construction of a putative UDT cDNA for isolation of *FcPT1*

Fig. S5 *In silico* feature analysis of FcPT polypeptides

Fig. S6 *In vitro* assay of FcPT2

Fig. S7 Enzymatic properties of FcPT1a

Fig. S8 5M7H:dimethylallyltransferase (DT) activity of FcPT1

Fig. S9 UV spectra of FC compounds in fig latexes

Fig. S10 Gene structures of UDTs and their relatives

Fig. S11 *In silico* analysis of fig C2’H candidates

Fig. S12 *In silico* analysis of angiosperm COSY homologues

Table S1 Primer list

Table S2 PT polypeptides used for *in silico* analyses

Table S3 Contigs belonging to the UbiA superfamily in the comparable RNA-seq libraries among different latex types

Table S4 PT genes used for gene structure analysis.

References in the supporting information

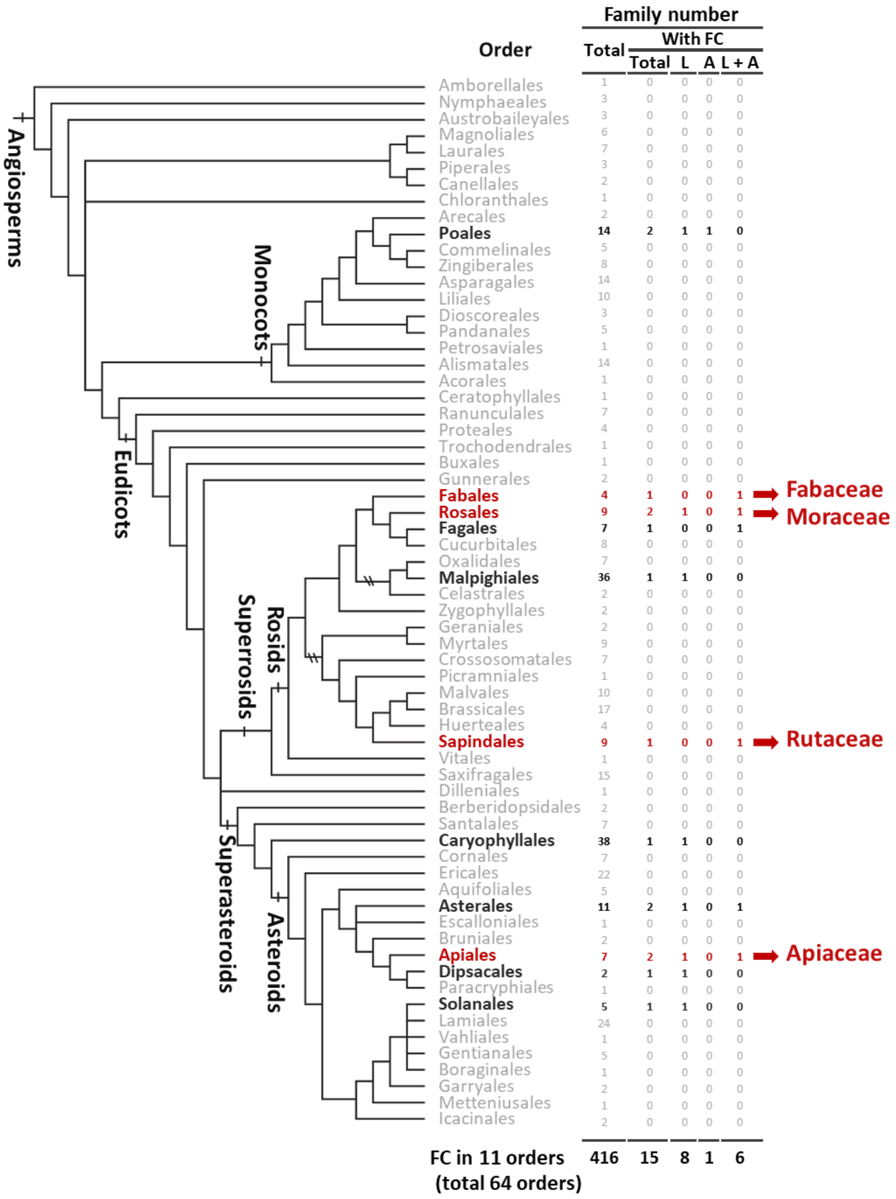

Fig. S1 Distribution of FCs in angiosperms

FC-containing plant species reported in Murray RDH (1982) are sorted according to the latest Angiosperm Phylogeny Group IV system (Murray *et al.*, 1982; Chase *et al.*, 2016). The total plant family number and the number of plant families containing FC molecules are shown for each plant order. Numbers under "L", "A", "L + A" indicate the numbers of plant families containing only linear, only angular, and both linear and angular FCs, respectively. The four major FC producers, *i.e.*, Apiaceae, Fabaceae, Moraceae, and Rutaceae, are classified in the "L + A" groups of Apiales, Fabales, Rosales, and Sapindales, respectively.

New Phytologist

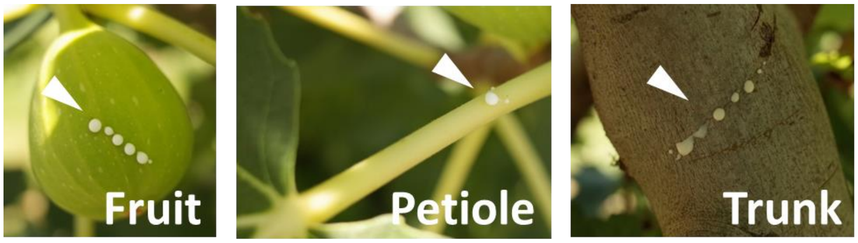

Fig. S2 Latexes from fig organs

Latexes exuded from cuts of fruits, petioles, and trunks of fig trees were used for this study.

(a)

```
>UDT-candidate1
GCACCAACAAGTTGACCCTGCCACCAGGGCTCTATGGTGAGAGCAGGTTCTCGAAACCCTTGCTGTTCGGCCA
ACATGACCGTAATTCCGCTCGGGCAAGCGCTCAAGCTCAAGCTGAAGGTTCTGATTCGGTACCACTTGCCAAC
AGTCAAGTTGGACTGTCCGTTCCCGATTCAGTGTTTGCCAAAGTTACACGTTTCTTATATACTTGCTATAAAT
TTGCAAGACCCTATGCCATGCGTCAATCAATTATATCGACTGTTTGTCTATATGCAAGAGTGTTGGTTGAAAA
TCCACAACTGTTTAAATGGTCTCTGTTGTTGAAGGCATTCCCTGGTCTAATTGCTGTATTCCTTGCATATACT
TACTATAATGGCACCAATCAGATCTTCGATATCGATATTGACAAGGTGAACAAACCGTATTTGCCTTTACCAG
CGGGAGAACTTACACTAAAACAAGCGTGGTACTTGGTGATATTCGATATAGTTGCTGGCCTGTCGATTTTGCG
GTTGATGAATGCAGACAAAATCACAACCATTTTATACTGCCTTGGTCTTTTCTTTTGCACACTCTACTCTGCT
CCTCCCTTTAGATTCAAGGGATCTTCTCTTGCAACAGTTATTGTGATTCCTTTGATCACTGGTGTCATTCAAA
ATATCGGTATACTTTATACCTCCCAAGTGTCTCTCGGACTTCCATTTTGGTGGAGCCCCCCAGTTGTTTTCAT
CACTGCCTTTAGTACATTGTTTTTTGTGATCATTAGCATCATCAAAGATATTCCCGACGTTGAAGGTGACTTA
AAGCATAACATTCGAACATTTGCGGCAATGTTCGGAGCGAAAAATATTGCAGTGGCTGCCATGGGACTGTTGT
TGATAAATTATCTTGCTCCTATAGCAGCTGCCTTTTACTTGCCTCAGGCTTTCAATCTTAAAGTGTTGCTGCC
CTCTCACTTAATCATGTCCGTTTGGTCAATATTTGAGTGGAGGAAGATGGTCAAAGAAAATTACAACCAGGAA
TCGAGCACAAATTTCTTTCAATTCCTTTGGACGCTTCTTATGATAGAGTTTTTATTGTTTCCTTTCATATAGA
AAAGTAGTGGTTTTTCCTTAACAGTGTAGAGAGAGTGAGGGAGGGACAAGAGTGGCGTG

>UDT-candidate2
AGGGGGGATTAAAGGGAAGAATTTGGATTAAAAAACCCTTTTTTTTTTTTTGGTGGTTGTTTTCAGTTCACAT
AGATGGAGCTCTCAATCTCTTACTCTTCTCTTAGACCATCAGCTGTTCTACCTCAACGTTGCAAGTCTTCCTC
TCATAACAATAGGATGATTTGCATTAAGCCTATAACCAAAAATATAAATAATTGTTCCTCAAACTTTCCATCA
AAATTATGCTCCACCAATAGATTGATCCAGCCTCTCGCGGGGCTCTATGGTGAAAGCAAGCAAGATAGGCCGC
GTAATATCATCCGGGCATGTGGTCGGGACGAACACGATGATTCCGCTTCATCGACGGGACTCACCAAAGTTTT
GCGTTTTGGATCTGCTTGCTATAAATTCGTAAGGCCTTATGCAATGCTCCATACGCTTATATCGACCATTTGT
TTATTTGCAAGAGTGTTAGTTGAGAATCCACAGTTGTTTACATGGTCTCTCCTGCTGAAAGCATTCCTTGGTC
TTATTGCCGTTTTGCTCTTGAATGCTTATTATTGTGGCCATAATGGGATTTACGATGCTGATATTGATAGGGT
GAACAAGCCGGATTTACCAATATCATCAGGAGATCTTTCGCTAAAACAAGCATGGTTTTTGGTGATATTTGCT
GTACTTTCTGGCTTGTTGATTTTACGATTGATGAACGCCGACCTCATCACTACTTCTTTATATTGTTTTTGGA
TTTTATTGGCCACCTCCTATTCTGCTCCTCCGTTCAGATTTAAGCAATCTTCCGTTGCAACATCTATTGTCAT
TCCTTTGATGGGCGGTATTGTTCACCTTGTCGGCGTACTTTATGCTACGAGAGCTTCTCTTGGACTTTCATTT
CAGTGGAGTCCTTCAACTATTTTTATCACCACCTTTGCGACACTATTTTTTCTAGTAATTTGCAACATAAAAG
ATCTCGCGGACGTGGAAGGTGATATCAAGTATAATATTCGAACATTTCCAGCTATATATGGACCTAGAAAAGT
TACAATCTTTTTCACTGGAATACTTCTACTAGATTACATTGGTTCTATGTTGGTTGCCATCTGCATGCCTCAG
GCTTTCAAACCTTACATAATGGCACCCGCTCATGCAATTTTCTCTTTGTGGATACTTATCGAGGCCAAGAAGT
TGGACAAAACAAATTATAGCAAGGAAGCAAGCACAAATTTCTTTCAACTTCTTTGGAAGCTTTTTGCGTTGGA
ATTTCTCTTGTTTCCTTTCATGTAGTTATTAGTGCAAATTATTATACTTTTCGTTAATTTATACTTTAGTGTA
GAGAGTATAACCATGAGAGTTGTTTTGGAATAATATTGTTACCATATTCTTGTTTTATTTCTTCAGTAAATTG
TTGGGGTTGTATGATTTTCAAA

>UDT-candidate3
TTTTTTTTTTTTTTGGTGGTTGTTTTCAGTTCACATAGATGGAGCTCTCAATCTCTTACTCTTCTCTTAGACC
ATCAGCTGTTCTACCTCAACGTTGCAAGTCTTCCTCTCATAACAATAGGATGATTTGCATTAAGCCTATAACC
AAAAATATAAATAATTGTTCCTCAAACTTTCCATCAAAATTATGCTCCACCAATAGATTGATCCAGCCTCTCG
CGGGGCTCTATGGTGAAAGCAAGCAAGATAGGCCGCGTAATATCATCCGGGCATGTGGTCGGGACGAACACGA
TGATTCCGCTTCATCGACGGGACTCACCAAAGTTTTGCGTTTTGGATCTGCTTGCTATAAATTCGTAAGGCCT
TATGCAATGCTCCATACGCTTATATCGACCATTTGTTTATTTGCAAGAGTGTTAGTTGAGAATCCACAGTTGT
TTACATGGTCTCTCCTGCTGAAAGCATTCCTTGGTCTTATTGCCGTTTTGCTCTTGAATGCTTATTATTGTGG
CCATAATGGGATTTACGATGCTGATATTGATAGGGTGAACAAGCCGGATTTACCAATATCATCAGGAGATCTT
TCGCTAAAACAAGCATGGTTTTTGGTGATATTTGCTGTACTTTCTGGCTTGTTGATTTTACGATTGATGAACG
CCGACCTCATCACTACTTCTTTATATTGTTTTTGGATTTTATTGGCCACCTCCTATTCTGCTCCTCCGTTCAG
ATTTAAGCAATCTTCTGTTGCAACATCTATTGTCATTCCTTTGATGGGCGGTATTGTTCACCTTGTCGGCGTA
CTTTATGCTACGAGAGCTTCTCTTGGACTTTCATTTCAGTGGAGTCCTTCAACTATTTTTATCACCACCTTTG
CGACACTATTTTTTCTAGTAATTTGCAACATAAAAGATCTCGCGGACGTGGAAGGTGATATCAAGTATAATAT
TCGAACATTTCCAGCTATATATGGACCTAGAAAAGTTACAATCTTTTTCACTGGAATACTTCTACTAGATTAC
ATTGGTTCTATGTTGGTTGCCATCTGCATGCCTCAGGCTTTCAAACCTTACATAATGGCACCCGCTCATGCAA
TTTTCTCTTTGTGGATACTTATCGAGGCCAAGAAGTTGGACAAAACAAATTATAGCAAGGAAGCAAGCACAAA
TTTCTTTCAACTTCTTTGGAAGCTTTTTGCGTTGGAATTTCTCTTGTTTCCTTTCATGTAGTTATTAGTGCAA
ATTATTATACTTTTCGTTAATTTATACTTTAGTGTAGAGAGTATAACCATGAGAGTTGTTTTGGAATAATATT
GTTCCAAAACAAC
```

(b)

```
>C2'H-candidate1
CTTGCACATAGCTTGTCGAGCACAACAGCCATAAGCTTCTCTCTTCATCTTCTTCTTAACCTACAAACTCTTA
AAACATATTCTTTATCAAGTTTAAGCTCTTTCAATCCTATCAATGGCGCCTTCCGAAATCGGTTACACCACCG
ATCATGTCACCGACTTTGTCATAAACAAAGGCAATGGTGCAAAGGGTCTCTCCGAAACCGGCATCAAAACCAT
CCCGAAACAGTATATCCAGCCTCTTGAAGAGAGGACCATGAACAAGGTCATGGTCGGGGAGTCTATTCCGATC
ATCGATGTCTCTAACTGGGACGATCCCAAAGTGCAGTCGGCCATTTGGGACGCTGCGGAGAAATGGGGTTTCT
TTCAGATCATCAACCATGGAGTGCCCATCGAAGTGCTTGACAACGTCAAAGAGGGGACATGTCGCTTCTTCGA
CTTGCCTGCTGAGGAGAAGAACAAGTATTCCAAAGAGAATACGGTGTCCAACAATGTCCGATACATCACCAGC
TTTATTCCTGAAGTTGAGAAGGCTTTGGAATGGAAAGATTACCTCAGTCTCTTTTATGTTTCTGAGGATGAGG
CCAATAGGCTGTGGCCTTCAGCTTGCAAGGATCAAGTTTTAGAATACATGAATCAGAGCGAAAAAGTTGTCAA
CCAATTGCTACGTGTGCTAATGGATGGGCTAAATGTGACAGAAATAGATAGTGAAAAAGAAAAGCTTCTGTCA
GGTTCAAGAAGGATTAACCTGAATTACTACCCTAAATGTCCGAGTCCGGAGCTTACAGTGGGAGTCGGTCGCC
ACTCCGATGTCTCCACATTTACCGTTCTTCTTCAAGATGATATCGGCGGGCTTTACGTGCGAGTTGAGGCTAG
AGACAGTTGGGTACATGTTCCGCCAGTAAAAGGGTCACTTGTTATCAACATAGGTGATGCACTGCAAATCTTC
AGTAACGGACGATATAGAAGCATTGAACACAGAGTGGCAGCTAACGGAGTGAGTGACAGGATCTCAGTTCCCA
TCTTTGTCAACCCAAGGCCAACCGACACCATCTCTCCCTTGCCTGAAGTGCTTGCTAATACCGGCCAGGAACC
GATCTACAAACCAGTTCTGTACTCAGATTATGTCAAGCACTTCTACAGAAAGGCCCACGATGGAAAGCTTACC
ATTGATTTCGCAAAGAAATAAGGCAATTTTATGTGTTGGATCTGATCAGAACAGAAACTTTTACTTCTGCAGA
TGAAAAACTAAATTACTTAATGATTTGTAAGTATCTGAAGATGCAGTGAGACGCTCTATGAAATAAATGTGAT
AATATGTAAGGCCAGTGAAAAAATTGTAATAATCTCTGATAATATTGGCTCTTAATGTGTTTTGTAAGAGATA
AATAATACTGTTGTTTGTACTCTTTTCTGTATGTACATATTATTTTGTTCTATAAAAATATATATACACTTTT
TTATCAAAAAAAAAAAAATCTTGATCAGCC
```

Fig. S3 Contigs of an RNA-seq prepared from fig fruit latexes

UDT candidates **(a)** and a C2'H candidate **(b)** identified by tblastn analyses of our RNA-seq library prepared from latexes of fig fruits. CDSs of these candidates are underlined.

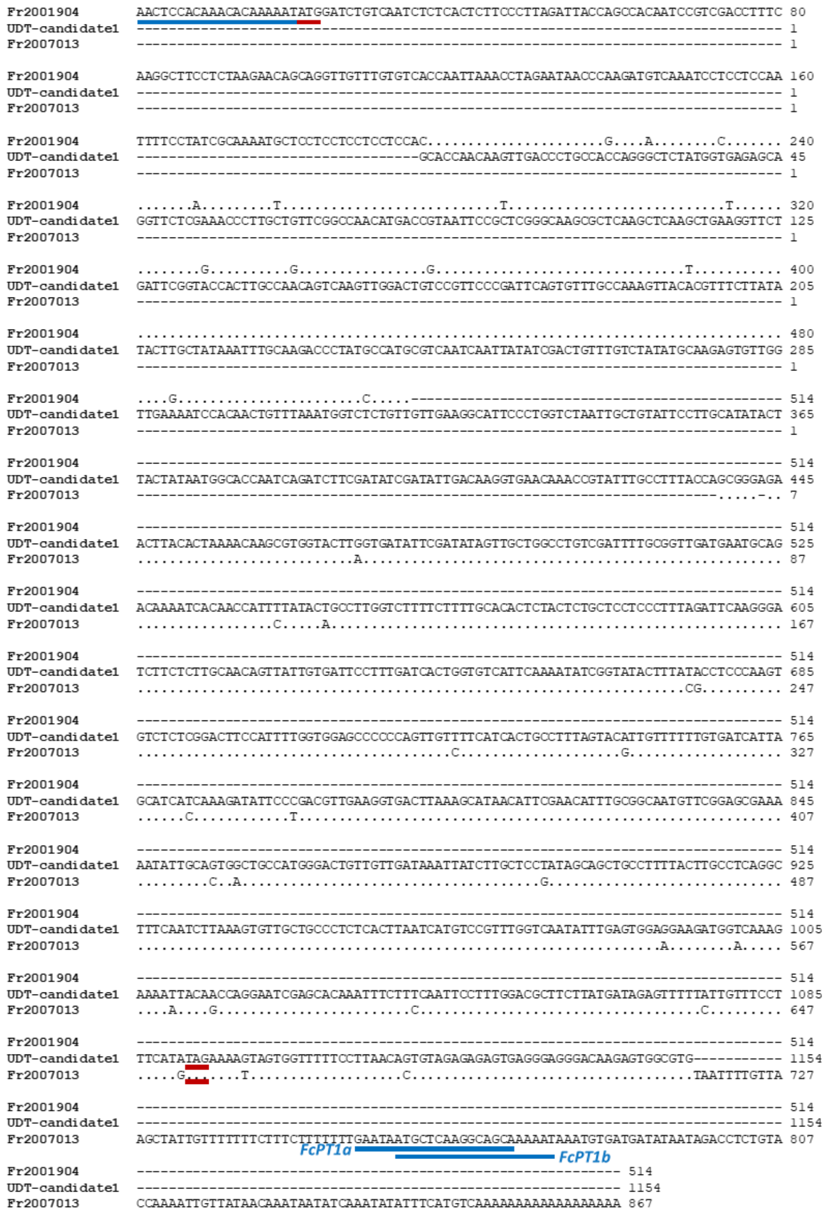

Fig. S4 *In silico* construction of a putative UDT cDNA for isolation of *FcPT1*

A *UDT* candidate contig (candidate 1, Fig S3a) from the fig fruit latex RNA-seq library and *F. religiose* contigs (Fr2001904 and Fr2007013) were aligned to construct a putative UDT cDNA sequence used to isolate the full CDS of *FcPT1*. Assessment of overlapping regions of the three sequences showed that the nucleotide sequence of candidate1 was 96% and 97% identical to Fr2001904 and Fr2007013, respectively. The start/stop codons of this *in silico* CDS and UTR regions for which primers were designed are underlined in red and blue, respectively.

New Phytologist

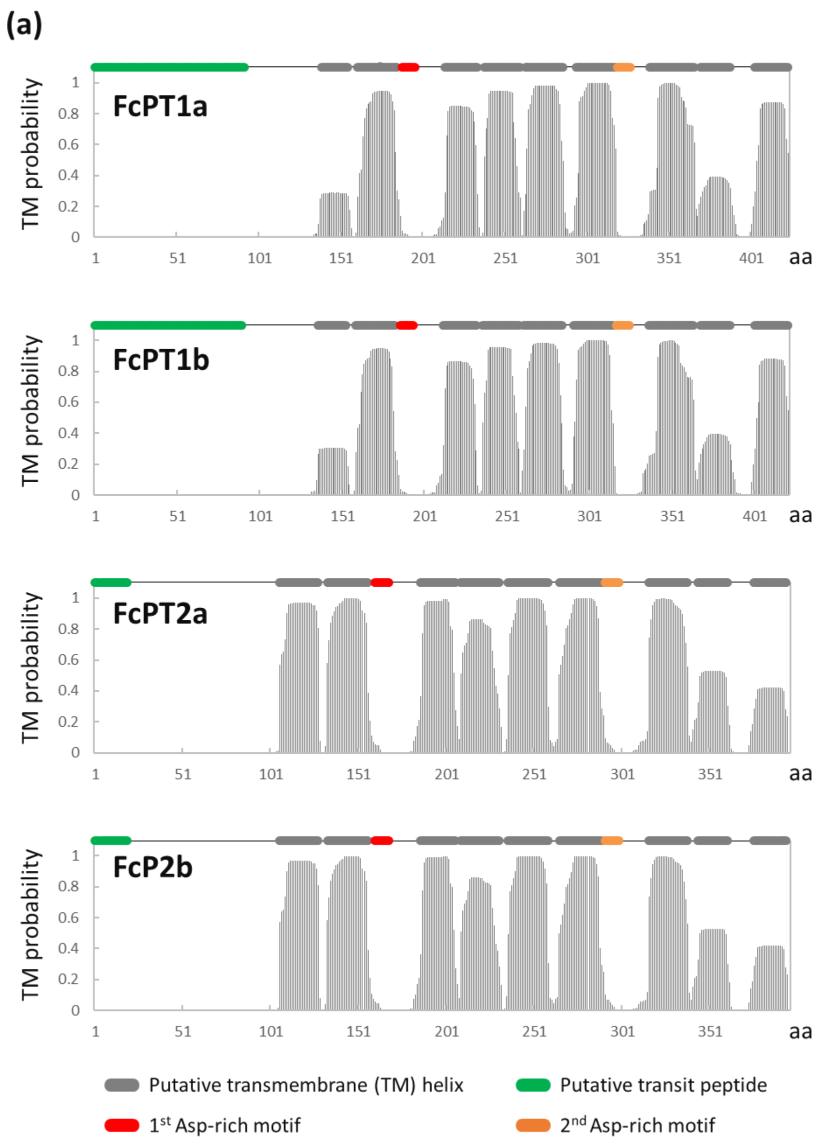

(b)

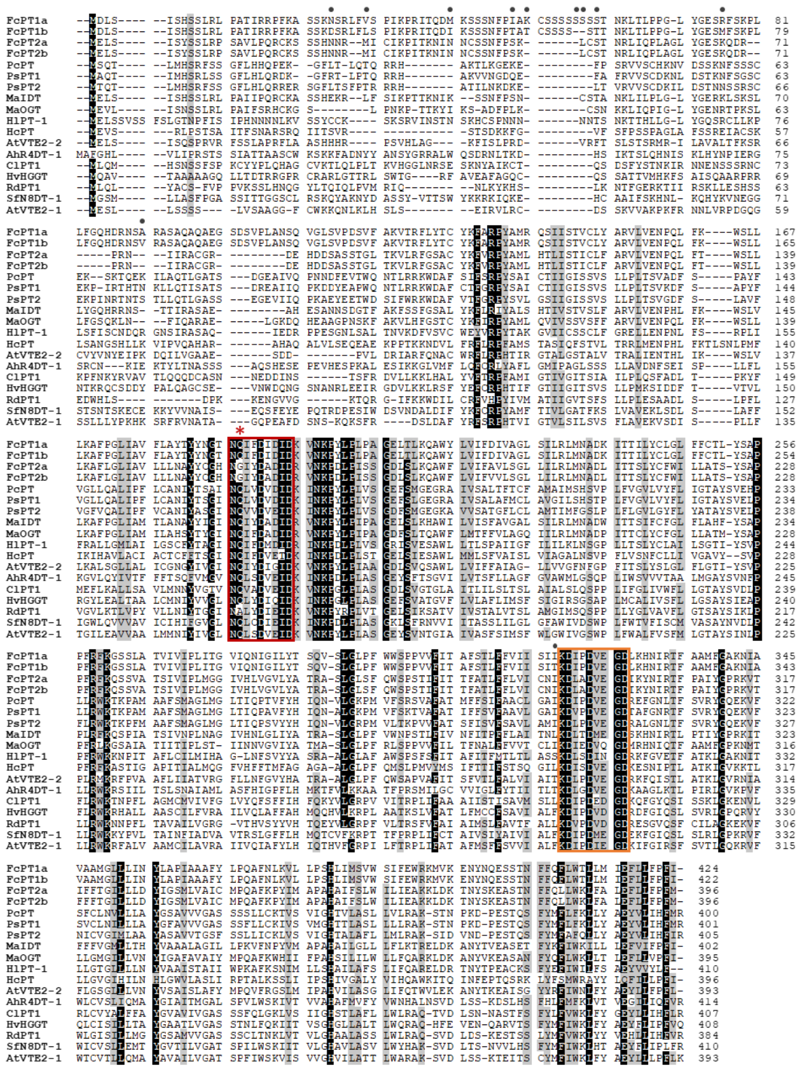

Fig. S5 *In silico* feature analysis of FcPT polypeptides

(a) Predicted structural features of the four FcPT polypeptides. Amino acids predicted to possess high transmembrane (TM) possibilities (>25%) by TMHMM are shown as components of TM helixes (grey bars) together with the N-terminal transit peptides predicted by ChloroP (green bars) and the first and second aspartate-rich motifs (red and orange bars, respectively).

(b) Multiple alignment of FcPT polypeptides and other related PT members. Red and orange boxes indicate the first and second aspartate-rich motifs, respectively. Circles and an asterisk indicate mismatches between FcPT1a/b and the locus in which FcPT2s possess an atypical glycine in the first aspartate-rich motif, respectively. The plant origins and accession numbers of PT members are listed in Table S2.

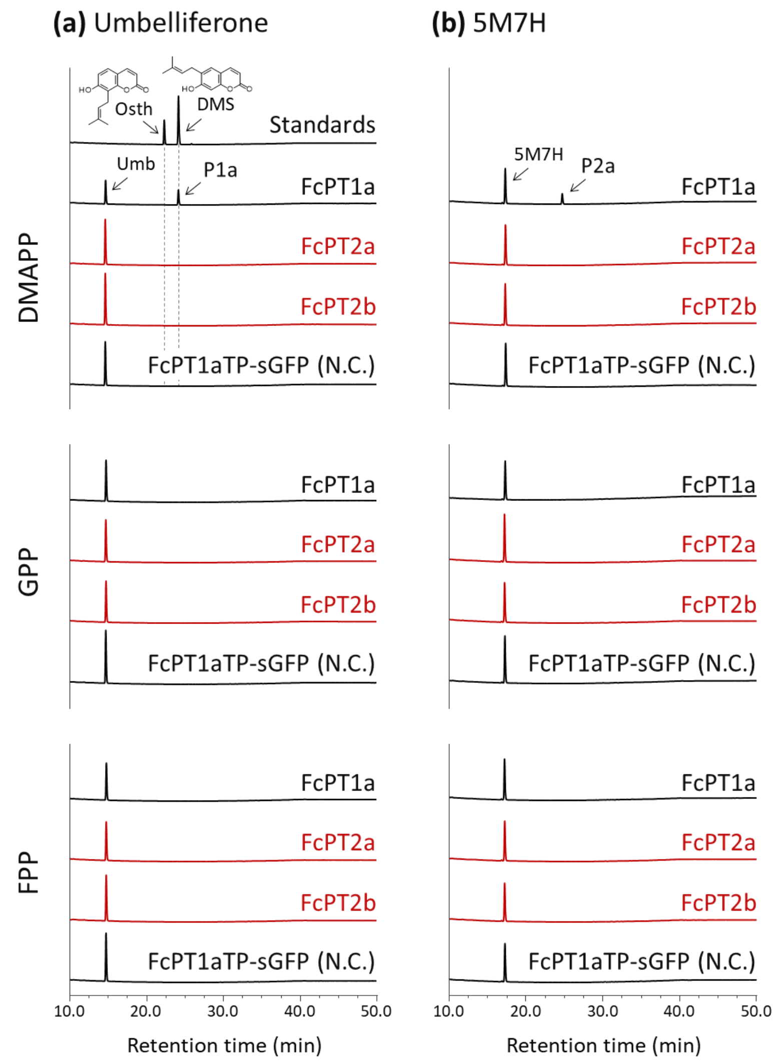

Fig. S6 ***In vitro*** **assay of FcPT2**

UV chromatograms of FcPT2 reaction mixtures. FcPT2a/b microsomes were incubated with umbelliferone (umb) **(a)** or 5-methoxy-7-hydroxycoumarin (5M7H) **(b)** as a prenyl acceptor and dimethylallyl diphosphate (DMAPP) (top), geranyl diphosphate (GPP) (middle) or farnesyl diphosphate (FPP) (bottom) as a prenyl donor in the presence of Mg^{2+} as a cofactor. FcPT1a and FcPT1aTP-sGFP (a negative control) microsomes from the same preparation lot were tested in parallel. HPLC analysis was performed using acetonitrile as the solvent instead of methanol, and UV monitoring was prolonged with solvent B composition kept at 99% after the gradient step. For each substrate combination, chromatograms were shown at 330 nm and at a comparable scale.

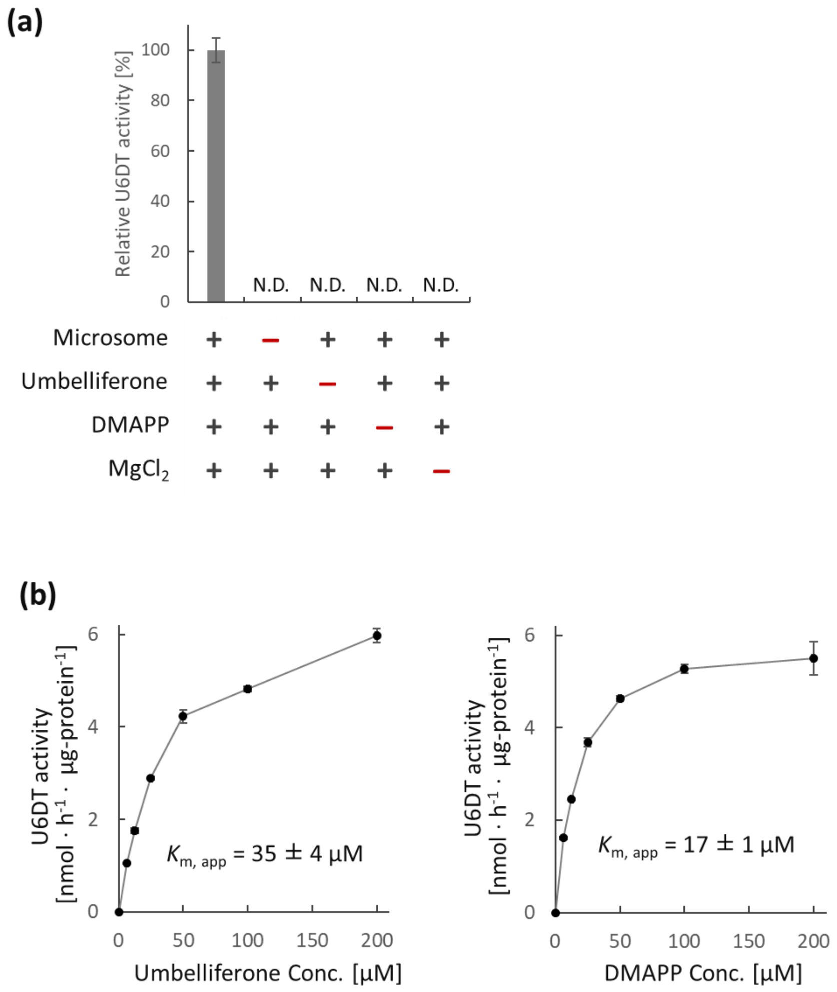

Fig. S7 Enzymatic properties of FcPT1a

(a) Different negative control assays. From left, full assay, negative controls with heat-denatured crude enzymes (microsomes boiled at 95 °C for 20 min), without umbelliferone or DMAPP, and with EDTA instead of $MgCl_2$. Values shown are means ± standard errors (n = 3 each). N.D., not detected.

(b) Affinities of FcPT1a for umbelliferone (left) and DMAPP (right). Microsomes were incubated for 2 h with 200 µM DMAPP and different concentrations of umbelliferone (0–200 µM) (left) or with 200 µM umbelliferone and different concentrations of DMAPP (0–200 µM) (right). Values shown are means ± standard errors (n = 3 each).

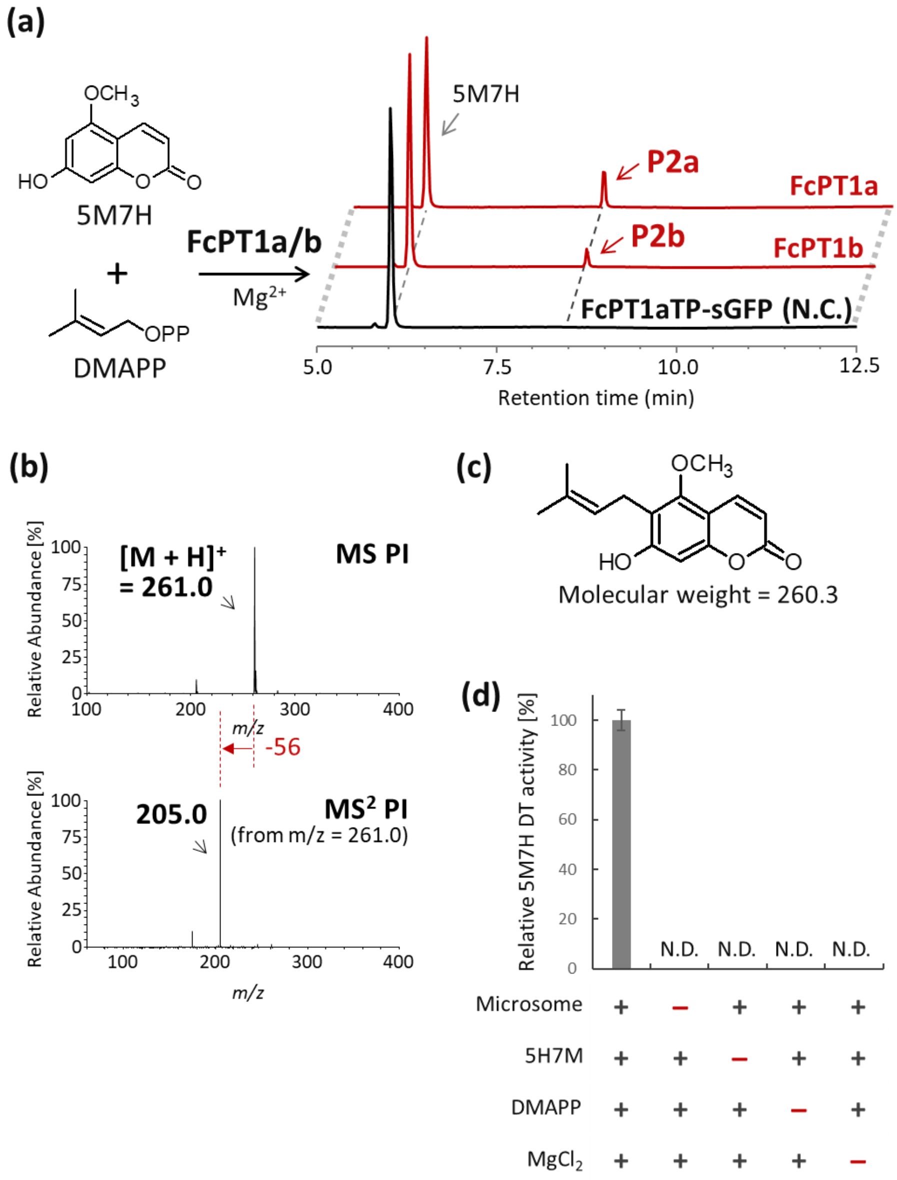
(a)
OCH3
HO
5M7H
+
DMAPP
OPP
FcPT1a/b
Mg2+
5M7H
P2a
FcPT1a
P2b
FcPT1b
FcPT1aTP-sGFP (N.C.)
5.0
7.5
10.0
12.5
Retention time (min)
(b)
Relative Abundance [%]
[M + H]+ = 261.0
MS PI
m/z
-56
205.0
MS2 PI
(from m/z = 261.0)
(c)
OCH3
HO
Molecular weight = 260.3
(d)
Relative 5M7H DT activity [%]
N.D.
Microsome
5H7M
DMAPP
MgCl2

Fig. S8 5M7H:dimethylallyltransferase (DT) activity of FcPT1

(a) UV chromatograms of 5M7H:DT reaction mixtures of FcPT1a/b. Microsomes from *N. benthamiana* expressing *FcPT1aTP-sGFP* were used as a negative control. All chromatograms are shown at 330 nm and at a comparable scale.

(b) MS and MS^2 spectra of P2a in the positive ion mode. The loss of 56 mass units represents specific fragmentation to dimethylallyl moieties attached to aromatic rings via C-C bonds (Simons *et al.*, 2009).

(c) A possible structure for the enzymatic product, P2.

(d) Different negative control assays. From left; full assay, negative controls with heat-denatured crude enzymes (microsomes boiled at 95 °C for 20 min), without 5M7H or DMAPP, and with EDTA instead of $MgCl_2$. Values are means ± standard errors (n = 3 each). N.D., not detected.

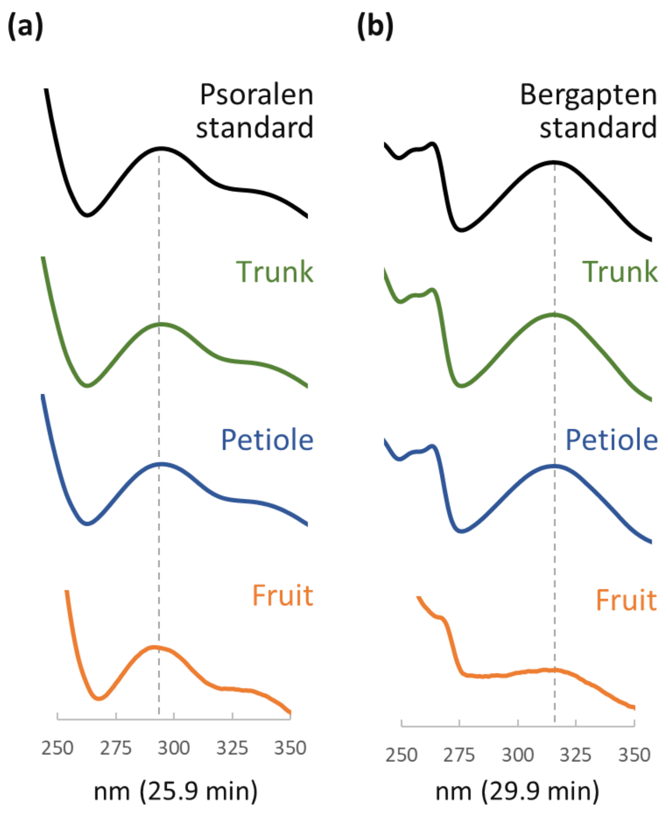

Fig. S9 UV spectra of FC compounds in fig latexes

UV spectra at the retention times of psoralen **(a)** and bergapten **(b)** in the chromatograms of Fig. 4a. From the top, the rows show a standard of psoralen or bergapten and methanol extracts of latexes from trunks, petioles, and fruits of fig.

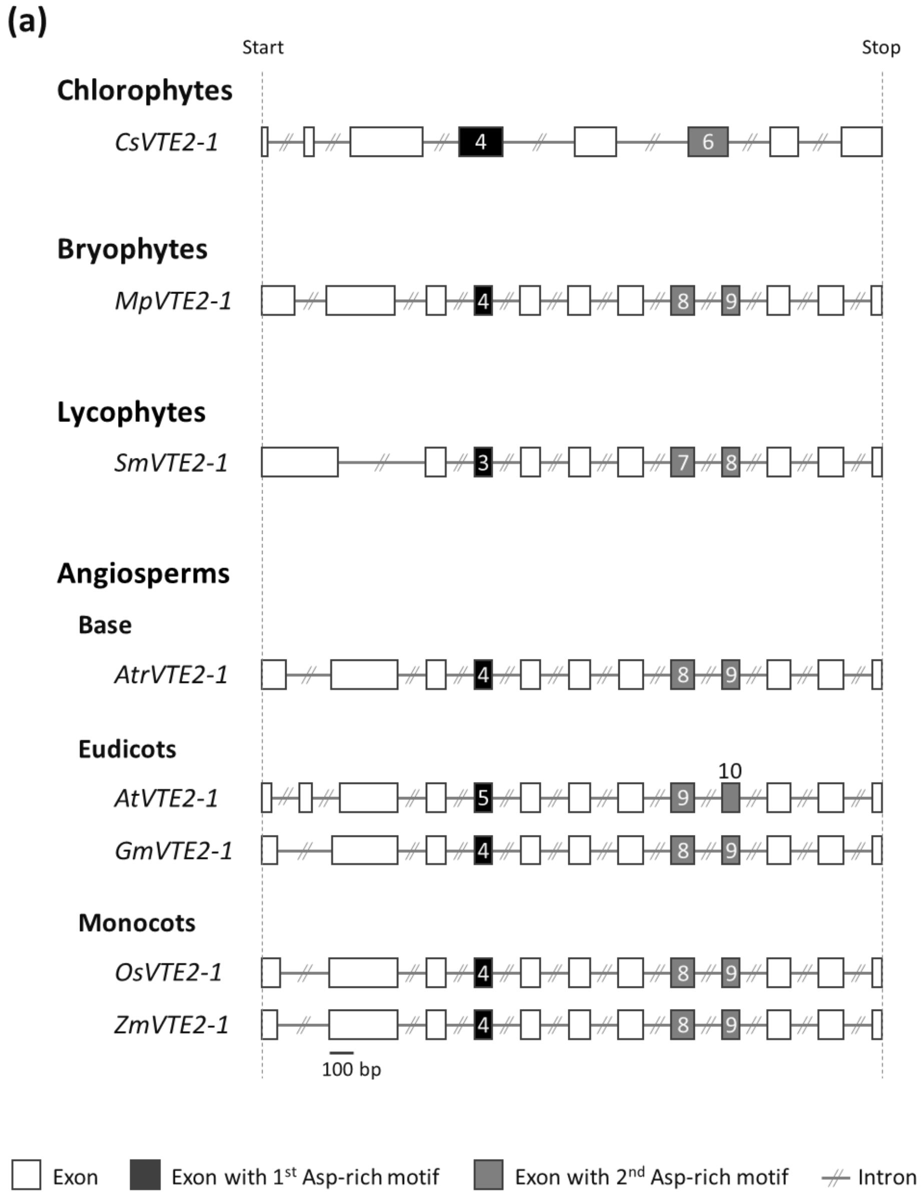

(a)
Start
Stop
Chlorophytes
CsVTE2-1
4
6
Bryophytes
MpVTE2-1
4
8
9
Lycophytes
SmVTE2-1
3
7
8
Angiosperms
Base
AtrVTE2-1
4
8
9
Eudicots
AtVTE2-1
5
9
10
GmVTE2-1
4
8
9
Monocots
OsVTE2-1
4
8
9
ZmVTE2-1
4
8
9
100 bp
Exon
Exon with 1st Asp-rich motif
Exon with 2nd Asp-rich motif
Intron

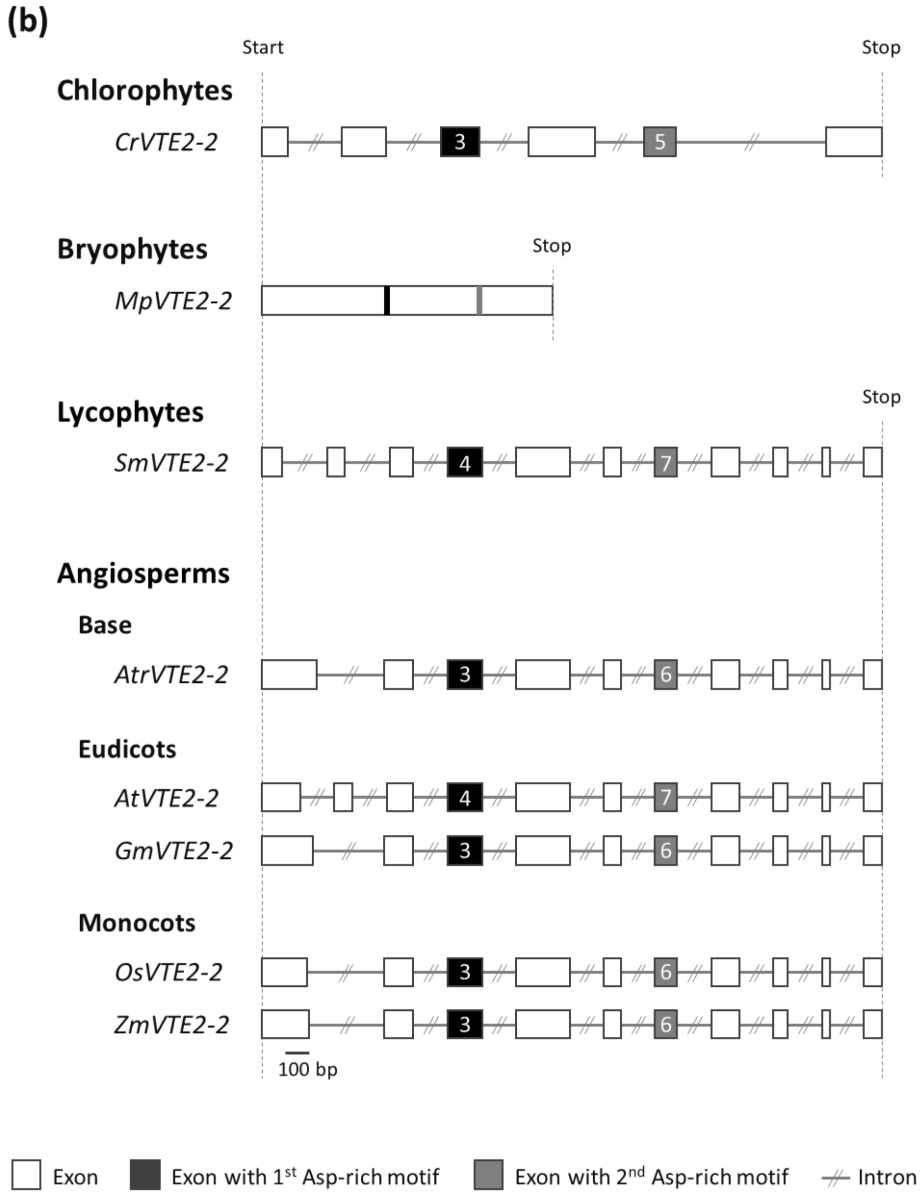
(b)
Start
Stop
Chlorophytes
CrVTE2-2
3
5
Bryophytes
Stop
MpVTE2-2
Lycophytes
Stop
SmVTE2-2
4
7
Angiosperms
Base
AtrVTE2-2
3
6
Eudicots
AtVTE2-2
4
7
GmVTE2-2
3
6
Monocots
OsVTE2-2
3
6
ZmVTE2-2
3
6
100 bp
Exon
Exon with 1st Asp-rich motif
Exon with 2nd Asp-rich motif
Intron

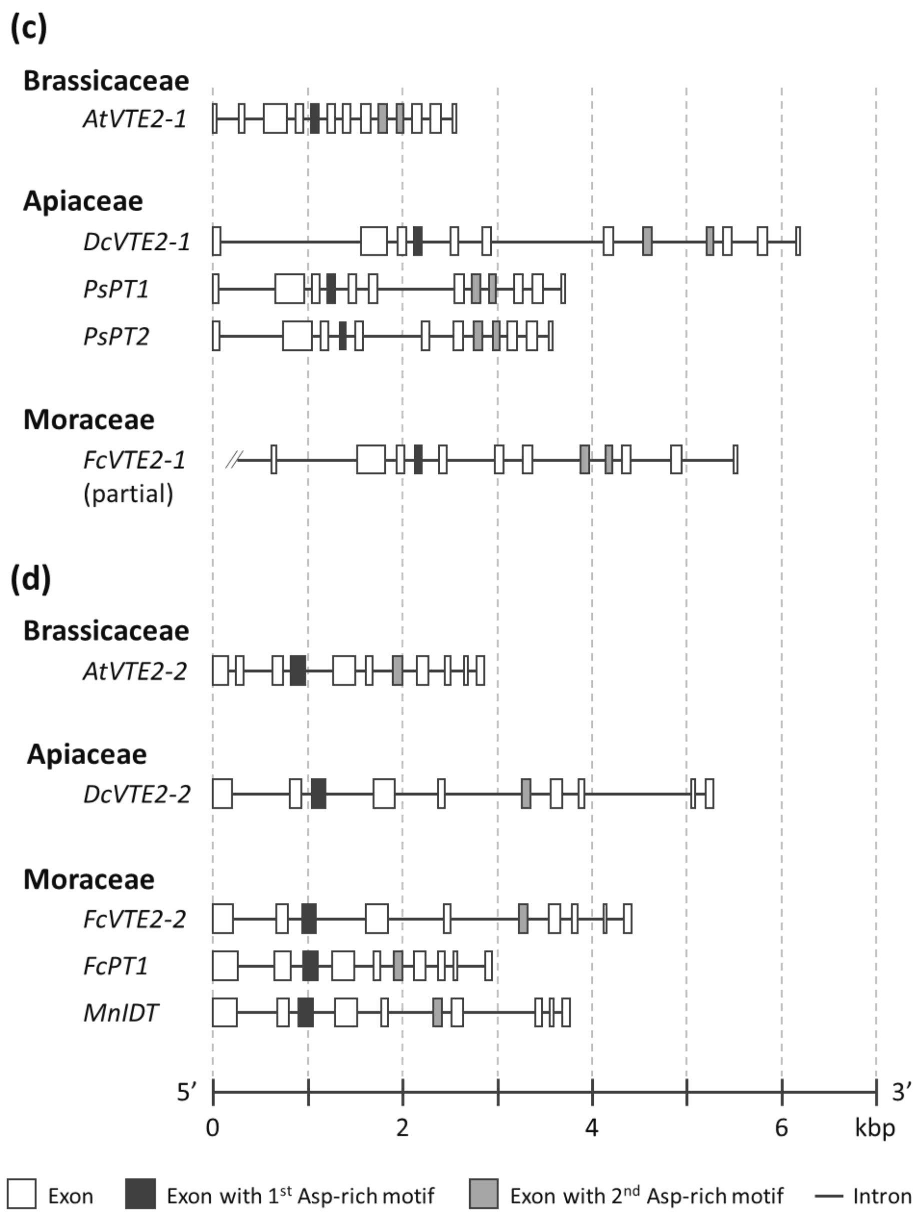

Fig. S10 Gene structures of UDTs and their relatives

Exon structures of *VTE2-1* **(a)** and *VTE2-2* **(b)** in various plant taxa. Exon-intron structures of Apiaceae *UDTs* **(c)** and *FcPT1* **(d)**, together with related *PT* genes. Exons containing the first and second aspartate-rich motifs shown in black and grey, respectively, and are numbered in **(a)** and **(b)**. As for MpVTE2-2, the positions of the first and second aspartate-rich motifs in the exon are shown by the same coloring. Detailed information for PT genes is shown in Table S4.

New Phytologist

(a)

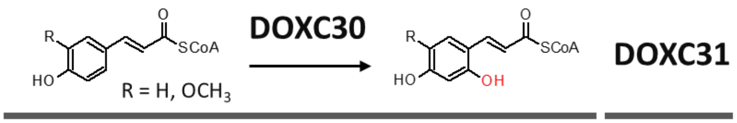

	PsC2'H	Ppc2'H	RgC2'H	IbF6'H/C2'H2-1-1	IbF6'H/C2'H2-1-8	IbF6'H/C2'H2-2-1	AtF6'H1	AtF6'H2	IbF6'H1-1	IbF6'H1-2	IbF6'H1-3	MeF6'H1	MeF6'H2	MeF6'H3	AtGSLOH	CrD4H	ZmBX6
37413_c0_g1_i2	50	49	53	51	51	52	54	49	53	53	54	57	57	52	27	35	32
37413_c0_g1_i4	50	48	53	52	51	52	55	49	53	53	54	57	57	51	27	29	32
37413_c0_g1_i5	50	49	53	51	51	52	54	49	53	53	54	57	57	52	27	35	32
37413_c0_g1_i6	51	52	54	55	55	55	55	53	56	56	56	56	56	54	30	32	31
37688_c0_g1_i1	53	53	56	56	55	56	56	54	57	57	58	58	58	56	29	32	31
37688_c0_g1_i2	50	48	53	52	51	52	55	49	53	53	54	57	57	51	27	29	32
37688_c0_g1_i3	50	49	53	51	51	52	54	49	53	53	54	57	57	52	27	35	32
37688_c0_g1_i11	50	48	53	52	51	52	55	49	53	53	54	57	57	51	27	29	32
40955_c4_g2_i1	61	61	62	69	68	68	65	66	70	70	70	70	70	71	29	34	34
40955_c4_g3_i1	60	57	64	55	54	56	64	62	63	63	64	63	63	60	29	28	29
40955_c4_g3_i2	59	57	63	54	53	56	63	62	61	61	63	61	61	58	29	28	30
40955_c4_g3_i3	59	56	65	56	55	56	65	63	63	63	64	59	60	59	28	29	29
40955_c4_g10_i1	64	64	65	69	69	69	67	65	72	72	72	68	68	69	29	34	34
40955_c4_g10_i2	71	70	68	75	75	75	71	69	73	74	74	68	67	69	33	38	40
40955_c4_g10_i3	70	69	69	75	75	75	71	70	75	75	75	69	69	70	34	40	42
40957_c0_g3_i1	61	56	64	54	52	54	63	60	59	59	61	54	55	54	31	31	29
40957_c0_g3_i2	61	58	63	53	52	55	63	60	59	59	60	58	58	55	32	30	29
40957_c0_g3_i3	63	60	65	62	61	62	65	63	66	66	67	64	64	63	31	34	33

0% – 100%

(b)

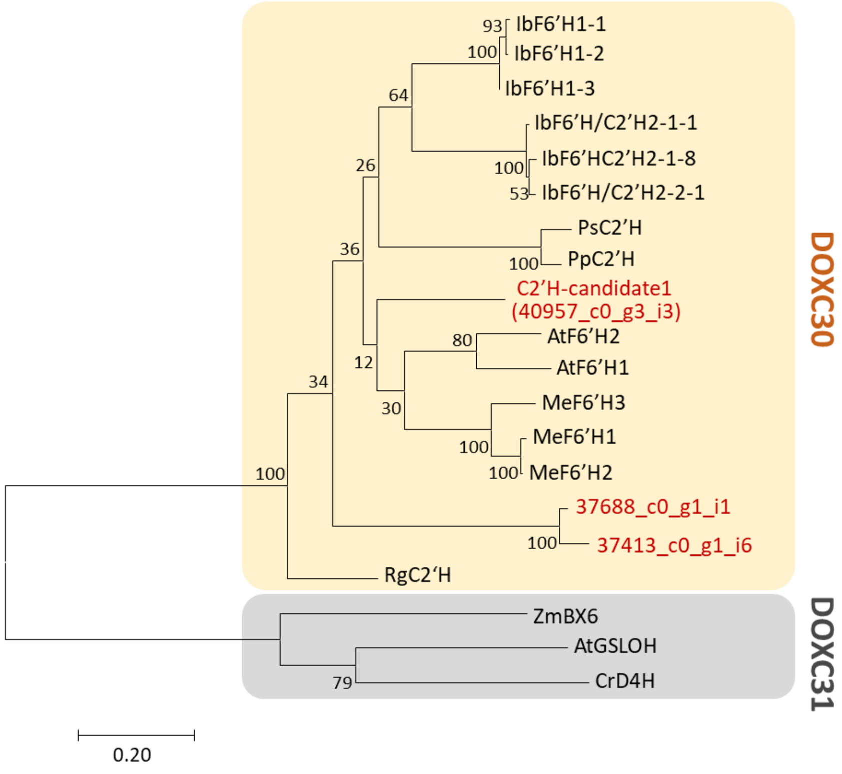

Fig. S11 ***In silico*** **analysis of fig C2’H candidates**

(a) Amino acid identities of the fig contigs previously annotated as C2’H (Kitajima *et al.*, 2018) relative to functionally identified DOXC30 members (C2’Hs and feruloyl CoA 6’-hydroxylases (F6’Hs)) and DOXC31 members (the closest outgroup functionally different from DOXC30s) (Kawai *et al.*, 2014). Among the candidates, 37413_c0_g1_i6, 37688_c0_g1_i1, and 40957_c0_g3_i3 were subjected to phylogenetic analysis because they encoded sufficiently long CDSs (37413_c0_g1_i6 and 37688_c0_g1_i1 contain full CDSs).

(b) Phylogenetic relationships among fig C2’H candidates and DOXC30/31 members. A maximum-likelihood phylogenetic tree, including the three C2’H candidates of full polypeptide sequences, is shown together with bootstrap values (maximum 100) by 1,000 bootstrap tests. Instead of 40957_c0_g3_i3 containing a partial CDS, C2’H-candidate1 encoding a full CDS almost identical to the partial sequence (amino acid identity 96%) is shown (Fig. S3b). The scale bar indicates an amino acid substitution rate per site of 0.2. Abbreviations for plant species are as follows: At, *Arabidopsis thaliana*; Cr, *Catharanthus roseus*; Ib, *Ipomoea batatas*; Me, *Manihot esculenta*; Pp, *Peucedanum praeruptorum*; Ps, *Pastinaca sativa*; Rg, *Ruta graveolens*; Zm, *Zea mays*.

Accession numbers of DIOX proteins are: AtF6’H1, NP_187970.1; AtF6’H2, NP_175925.1; AtGSLOH, NP_180115.1; CrD4H, O04847.2; IbF6’H1-1, G9M9M0.1; IbF6’H1-2, G9M9M1.1; IbF6’H1-3, G9M9M2.1; IbF6’H/C2’H2-1-1, G9M9M4.1; IbF6’H/C2’H2-1-8, G9M9M5.1; IbF6’H/C2’H2-2-1, G9M9M3.1; MeF6’H1, ARX98188.1; MeF6’H2, ARX98187.1; MeF6’H3, ARX98186.1; PpC2’H, ASR80916.1; PsC2’H, APP94171.1; RgC2’H, W5QJZ5.1; ZmBX6, NP_001105100.1.

(a)

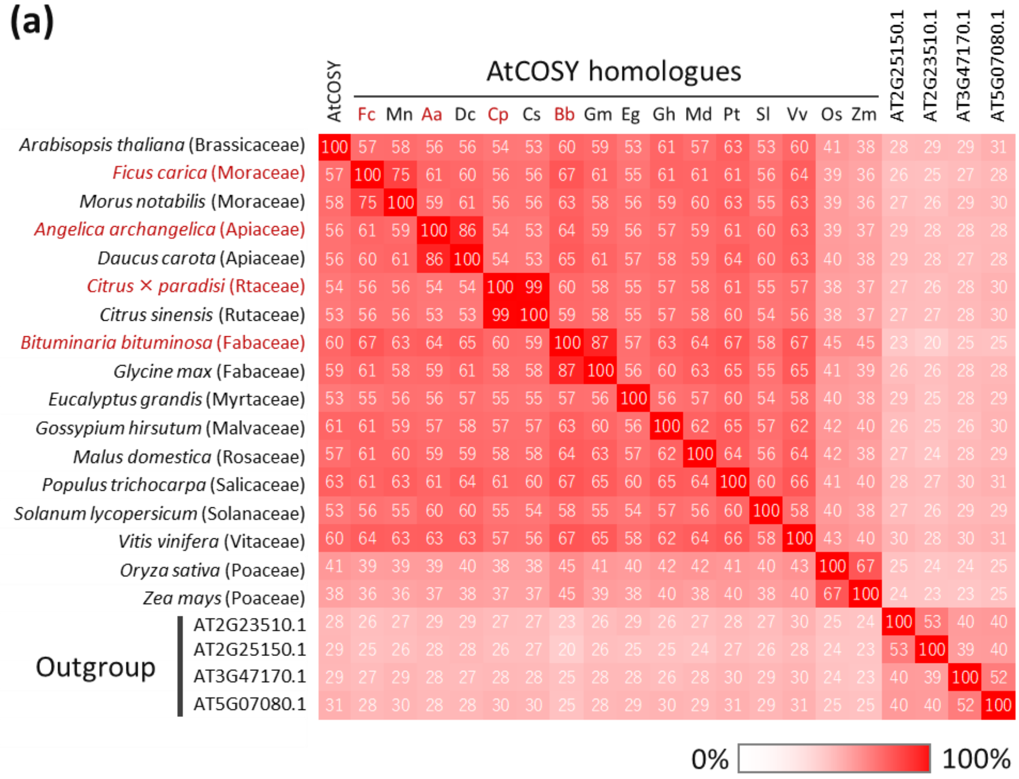
AtCOSY homologues
AtCOSY Fc Mn Aa Dc Cp Cs Bb Gm Eg Gh Md Pt Sl Vv Os Zm AT2G25150.1 AT2G23510.1 AT3G47170.1 AT5G07080.1
Arabisopsis thaliana (Brassicaceae) 100 57 58 56 56 54 53 60 59 53 61 57 63 53 60 41 38 28 29 29 31
Ficus carica (Moraceae) 57 100 75 61 60 56 56 67 61 55 61 61 61 56 64 39 36 26 25 27 28
Morus notabilis (Moraceae) 58 75 100 59 61 56 56 63 58 56 59 60 63 55 63 39 36 27 26 29 30
Angelica archangelica (Apiaceae) 56 61 59 100 86 54 53 64 59 56 57 59 61 60 63 39 37 29 28 28 28
Daucus carota (Apiaceae) 56 60 61 86 100 54 53 65 61 57 58 59 64 60 63 40 38 29 28 27 28
Citrus × paradisi (Rtaceae) 54 56 56 54 54 100 99 60 58 55 57 58 61 55 57 38 37 27 26 28 30
Citrus sinensis (Rutaceae) 53 56 56 53 53 99 100 59 58 55 57 58 60 54 56 38 37 27 27 28 30
Bituminaria bituminosa (Fabaceae) 60 67 63 64 65 60 59 100 87 57 63 64 67 58 67 45 45 23 20 25 25
Glycine max (Fabaceae) 59 61 58 59 61 58 58 87 100 56 60 63 65 55 65 41 39 26 26 28 28
Eucalyptus grandis (Myrtaceae) 53 55 56 56 57 55 55 57 56 100 56 57 60 54 58 40 38 29 25 28 29
Gossypium hirsutum (Malvaceae) 61 61 59 57 58 57 57 63 60 56 100 62 65 57 62 42 40 26 25 26 30
Malus domestica (Rosaceae) 57 61 60 59 59 58 58 64 63 57 62 100 64 56 64 42 38 27 24 28 29
Populus trichocarpa (Salicaceae) 63 61 63 61 64 61 60 67 65 60 65 64 100 60 66 41 40 28 27 30 31
Solanum lycopersicum (Solanaceae) 53 56 55 60 60 55 54 58 55 54 57 56 60 100 58 40 38 27 26 29 29
Vitis vinifera (Vitaceae) 60 64 63 63 63 57 56 67 65 58 62 64 66 58 100 43 40 30 28 30 31
Oryza sativa (Poaceae) 41 39 39 39 40 38 38 45 41 40 42 42 41 40 43 100 67 25 24 24 25
Zea mays (Poaceae) 38 36 36 37 38 37 37 45 39 38 40 38 40 38 40 67 100 24 23 23 25
Outgroup
AT2G23510.1 28 26 27 29 29 27 27 23 26 29 26 27 28 27 30 25 24 100 53 40 40
AT2G25150.1 29 25 26 28 28 26 27 20 26 25 25 24 27 26 28 24 23 53 100 39 40
AT3G47170.1 29 27 29 28 27 28 28 25 28 28 26 28 30 29 30 24 23 40 39 100 52
AT5G07080.1 31 28 30 28 28 30 30 25 28 29 30 29 31 29 31 25 25 40 40 52 100
0%
100%

(b)

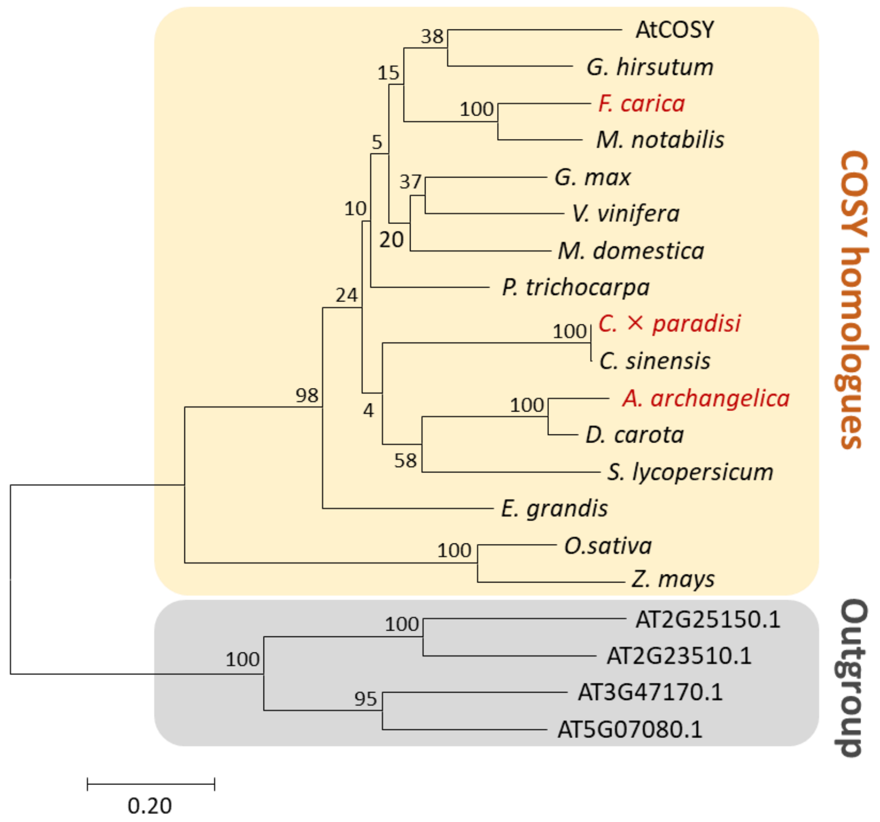
AtCOSY
G. hirsutum
F. carica
M. notabilis
G. max
V. vinifera
M. domestica
P. trichocarpa
C. × paradisi
C. sinensis
A. archangelica
D. carota
S. lycopersicum
E. grandis
O.sativa
Z. mays
AT2G25150.1
AT2G23510.1
AT3G47170.1
AT5G07080.1
38
15
100
5
37
10
20
24
100
98
4
100
58
100
100
100
95
COSY homologues
Outgroup
0.20

Fig. S12 *In silico* analysis of angiosperm COSY homologues

(a) Amino acid identities among *Arabidopsis thaliana* COSY (AtCOSY) and its homologues in angiosperms. The homologues are shown by the species name. The four closest *A. thaliana* homologues of AtCOSY are used as outgroup samples. COSY homologues of FC-rich species highlighted by red were screened by tblastn analysis of RNA-seq libraries using AtCOSY as a query. Protein sequences except for those from FC-rich species were selected in reference to the previous analysis (Vanholme *et al.*, 2019). For the *B. bituminosa* homologue lacking its N-terminal region, amino acid identities are calculated based on a ClustalW alignment corresponding to the partial region where the *B. bituminosa* sequence covers.

(b) A maximum-likelihood phylogenetic tree among AtCOSY and its homologues in angiosperms. All protein sequences analyzed in **(a)** except for the partial *B. bituminosa* homologue are used for the phylogenetic tree construction. The tree is shown together with bootstrap values (maximum 100) by 1,000 bootstrap tests. The scale bar indicates an amino acid substitution rate per site of 0.2.

Accession numbers of COSY homologues and outgroup proteins are: AtCOSY, NP_174189.1; *C. sinensis*, KDO77799.1; *Daucus carota*, XP_017220052.1; *E. grandis*, XP_010052232.1; *G. max*, XP_003541712.1; *G. hirsutum*, XP_016686828.1; *M. domestica*, XP_008383652.1; *M. notabilis*, XP_024029268.1; *O. sativa*, BAX24637.1; *P. trichocarpa*, XP_002305089.1; *S. lycopersicum*, XP_004238204.1; *V. vinifera*, XP_002271913.1; *Z. mays*, XP_020399988.1; AT2G23510.1, NP_179932.1; AT2G25150.1, NP_180087.1; AT3G47170.1, NP_190301.2; AT5G07080.1, NP_196325.1. COSY homologues of *C.* × *paradisi* (UHJR_scaffold_2011457), *A. archangelica* (TQKZ_scaffold_2061394), and *B. bituminosa* (TVSH_scaffold_2012425) were collected from RNA-seq libraries deposited in OneKP database. Fig COSY homologue is encoded by 6688_c0_g1_i1 in the fig latex RNA-seq libraries (Kitajima *et al.*, 2018).

New Phytologist

Table S1 Primer list

Primer name	Sequence (5'–3')
FcPT1_5'UTR_Fw	AACTCCACAAACACAAAAAT
FcPT1a_3'UTR_Rv	TGCTGCCTTGAGCATTATTC
FcPT1b_3'UTR_Rv	ATTTTTGCTGCCTTGAGCAT
FcPT2_5'UTR_Fw	GGTGGTTGTTTTCAGTTCACA
FcPT2a_3'UTR_Rv	TTCCAAAACAACTCTCATGGT
FcPT2b_3'UTR_Rv	TGAAAATCATACAACCCCAACA
FcPT1_TOPO_Fw	CACCATGGATCTCTCAATCTCTCAC
FcPT1_TOPO_Rv	CTATATGAAAGGAAACAATA
FcPT2_TOPO_Fw	CACCATGGAGCTCTCAATCTCTTACTC
FcPT2_TOPO_Rv	CTACATGAAAGGAAACAAGAG
FcPT1_TP_Rv	ATAGAGCCCTGGTGGCAGGG
FcPT1_qPCR_Fw	ATTCGGTACCACTTGCCAAC
FcPT1_qPCR_Rv	ATTGATTGACGCATGGCATA
FcActin_qPCR_Fw	ATGAAGGAGAAGCTGGCGTA
FcActin_qPCR_Rv	ACCTGTCCATCTGGCAATTC
PsPT2_BAC_Fw	ACATGGGATAGTATTTTCTGGAGAAAATGGG
PsPT2_BAC_Rv	CTTGTTAATCTGATAAACATACGCATCAAAGGTG

Table S2 PT polypeptides used for *in silico* analyses

(a) PT members involved in primary metabolism

PT member	Plant species	Accession ID
**Chrolophyll biosynthesis**		
AtATG4	*Arabidopsis thaliana*	NP_190750.1
GmATG4	*Glycine max*	NP_001239633.1
OsATG4	*Oryza sativa*	ABO31092.1
ZmATG4	*Zea mays*	NP_001142204.1
**Haem *a* biosynthesis**		
AtCOX10	*Arabidopsis thaliana*	NP_566019.1
GmCOX10	*Glycine max*	XP_003556552.1
OsCOX10	*Oryza sativa*	EEC70799.1
ZmCOX10	*Zea mays*	AFW89544.1
**Phylloquinone biosynthesis**		
AtABC4	*Arabidopsis thaliana*	NP_001117518.1
GmABC4	*Glycine max*	XP_003532605.1
OsABC4	*Oryza sativa*	NP_001049226.1
ZmABC4	*Zea mays*	NP_001152170.1
**Plastoquinone biosynthesis**		
AtVTE2-2	*Arabidopsis thaliana*	NP_001154609.1
AtrVTE2-2	*Amborella trichopoda*	XP_011628799.1
CrVTE2-2	*Chlamydomonas reinhardtii*	CAL01105.1
DcVTE2-2	*Daucus carota*	XP_017246707.1
FcVTE2-2	*Ficus carica*	BDEM01000270.1; 1
GmVTE2-2	*Glycine max*	KRH71769.1
MpVTE2-2	*Marchantia polymorpha*	Mapoly0082s0041.1
OsVTE2-2	*Oryza sativa*	XP_015646905.1
SmVTE2-2	*Selaginella moellendorffii*	XP_002979232.2
ZmVTE2-2	*Zea mays*	NP_001146703.1

Table S2 PT polypeptides used for *in silico* analyses

(a) PT members involved in primary metabolism – *continued*

PT member	Plant species	Accession ID
**Tocopherol biosynthesis**		
AtVTE2-1	*Arabidopsis thaliana*	NP_849984.1
AtrVTE2-1	*Amborella trichopoda*	XP_011623844.1
CsVTE2-1	*Coccomyxa subellipsoidea*	XP_005644040.1
DcVTE2-1	*Daucus carota*	XP_017253952.1
FcVTE2-1	*Ficus carica*	36048_c2_g1_i1
GmVTE2-1	*Glycine max*	NP_001241496.1
MpVTE2-1	*Marchantia polymorpha*	Mapoly0042s0068.1
OsVTE2-1	*Oryza sativa*	XP_015644510.1
SmVTE2-1	*Selaginella moellendorffii*	XP_002985546.2
ZmVTE2-1	*Zea mays*	ACG45339.1
**Ubiquinone biosynthesis**		
AtPPT1	*Arabidopsis thaliana*	NP_567688
GmPPT	*Glycine max*	XP_006602724.1
OsPPT1	*Oryza sativa*	BAE96574.1
ZmPPT	*Zea mays*	NP_001148558.1

FcVTE2-1 and FcVTE2-2 are collected in a fig RNA-seq library (Kitajima *et al.*, 2018) and a fig draft genome, respectively (Mori *et al.*, 2017). MpVTE2-1 and MpVTE2-2 were obtained from the Phytozome 12.1.6 database. The other sequences were derived from NCBI.

(b) VTE2-1-related members involved in specialized metabolism

PT member	Plant species	Accession ID
**Apiaceae**		
PcPT	*Petroselinum crispum*	BAO31627.1
PsPT1	*Pastinaca sativa*	AJW31563.1
PsPT2	*Pastinaca sativa*	AJW31564.1
**Ericaceae**		
RdPT1	*Rhododendron dauricum*	LC381857
**Fabaceae**		
AhR3'DT-1	*Arachis hypogaea*	AQM74173.1
AhR3'DT-2	*Arachis hypogaea*	AQM74174.1
AhR3'DT-3	*Arachis hypogaea*	AQM74175.1
AhR3'DT-4	*Arachis hypogaea*	AQM74176.1
AhR4DT-1	*Arachis hypogaea*	AQM74172.1
GmC4DT	*Glycine max*	BAW32575.1
GmG2DT	*Glycine max*	BAW32578.1
GmG4DT	*Glycine max*	NP_001235990
GmIDT1	*Glycine max*	BAW32576.1
GmIDT2	*Glycine max*	BAW32577.1
GmIDT3	*Glycine max*	XP_014618511.1
GmPT01	*Glycine max*	KRH76147.1
GuA6DT	*Glycyrrhiza uralensis*	AIT11912.1
GuILDT	*Glycyrrhiza uralensis*	AMR58303.1
LaPT1	*Lupinus albus*	AER35706.1
LjG6DT	*Lotus japonicus*	ARV85585.1
PcM4DT	*Psoralea corylifolia.*	AYV64464.1
SfFPT	*Sophora flavescens*	AHA36633.1
SfG6DT	*Sophora flavescens*	BAK52291.1
SfiLDT	*Sophora flavescens*	BAK52290.1
SfN8DT-1	*Sophora flavescens*	BAG12671.1
SfN8DT-2	*Sophora flavescens*	BAG12673.1
SfN8DT-3	*Sophora flavescens*	BAK52289.1
**Polaceae**		
HvHGGT	*Hordeum vulgare*	AAP43911.1
OsHGGT	*Oryza sativa*	AAP43913.1
TaHGGT	*Triticum aestivum*	AAP43912.1
ZmHGGT	*Zea mays*	XP_008659772.1
**Rutaceae**		
ClPT1	*Citrus limon*	BAP27988.1

(c) VTE2-2 and PPT-related members involved in specialized metabolism

PT member	Plant species	Protein ID
**Cannabaceae**		
CsPT3	*Cannabis sativa*	DAC76713.1
CsPT4	*Cannabis sativa*	DAC76710.1
HlPT-1	*Humulus lupulus*	BAJ61049.1
HlPT-2	*Humulus lupulus*	AJD80255.1
**Hypericaceae**		
HcPT	*Hypericum calycinum*	ALD84371.1
HcPT8px	*Hypericum calycinum*	AZK16226.1
HcPTpat	*Hypericum calycinum*	AZK16227.1
HsPT8px	*Hypericum sampsonii*	AZK16224.1
HsPTpat	*Hypericum sampsonii*	AZK16225.1
**Moraceae**		
CtIDT	*Cudrania tricuspidata*	AJD80983.1
MaIDT	*Morus alba*	AJD80982.1
MaOGT	*Morus alba*	AXN57307.1
**Boraginaceae**		
AePGT	*Arnebia euchroma*	ABD59796.2
AePGT4	*Arnebia euchroma*	ANC67957.1
AePGT6	*Arnebia euchroma*	ANC67959.1
LePGT1	*Lithospermum erythrorhizon*	BAB84122.1
LePGT2	*Lithospermum erythrorhizon*	BAB84123.1

New Phytologist

Table S3 Contigs belonging to the UbiA superfamily in the comparable RNA-seq libraries of different latex types

(a)

	Contig	Query with highest homology	Amino acid identity (%)	Predicted function
*	FcPT1a	FcPT1a	100	U6DT
*	FcPT1b	FcPT1b	100	U6DT
*	18727_c0_g1_i2	FcPT1a/b	64	Unknown
*	29478_c0_g1_i1	FcPT1a/b	99	U6DT
*	29478_c0_g1_i3	FcPT1a/b	99	U6DT
*	31647_c0_g1_i2	FcPT1b	52	Unknown
*	31647_c0_g1_i3	FcPT1a	57	Unknown
*	35740_c0_g3_i1	FcPT1a/b	100	U6DT
*	35740_c0_g3_i4	FcPT1a/b	100	U6DT
*	36524_c2_g1_i2	FcPT1a/b	64	Unknown
*	36524_c2_g1_i3	FcPT1a/b	64	Unknown
*	36524_c3_g1_i1	FcPT1b	100	U6DT
*	36524_c3_g2_i1	FcPT1a	51	Unknown
*	36524_c3_g2_i2	FcPT1a	55	Unknown
*	36524_c3_g2_i3	FcPT1a	55	Unknown
	37574_c0_g1_i1	FcPT1b	52	Unknown
	40931_c3_g1_i1	FcPT1b	44	Unknown
	40931_c3_g1_i2	FcPT1b	44	Unknown
	69328_c0_g1_i1	FcPT1a	58	Unknown
	38680_c0_g2_i6	AtPPT	39	Unknown

(b)

Contig	Query with highest homology	Amino acid identity (%)	Predicted function
31512_c0_g1_i1	AtABC4	68	ABC4
31512_c0_g1_i2	AtABC4	68	ABC4
31512_c0_g1_i3	AtABC4	68	ABC4
31512_c0_g1_i4	AtABC4	68	ABC4
36921_c0_g2_i1	AtABC4	75	ABC4
36921_c0_g2_i3	AtABC4	68	ABC4
36921_c0_g2_i5	AtABC4	75	ABC4
36921_c0_g2_i6	AtABC4	72	ABC4
27273_c0_g2_i2	AtATG4	100	ATG4
30180_c1_g1_i1	AtATG4	80	ATG4
30180_c1_g1_i2	AtATG4	80	ATG4
34939_c0_g1_i1	AtATG4	79	ATG4
34939_c0_g1_i2	AtATG4	76	ATG4
34939_c0_g1_i3	AtATG4	80	ATG4
34939_c0_g1_i5	AtATG4	76	ATG4
34939_c0_g1_i7	AtATG4	80	ATG4
39363_c0_g5_i2	AtCOX10	64	COX10
39363_c0_g5_i3	AtCOX10	64	COX10
39363_c0_g5_i4	AtCOX10	64	COX10
39363_c0_g5_i5	AtCOX10	63	COX10
39363_c0_g5_i6	AtCOX10	63	COX10
39363_c0_g5_i7	AtCOX10	63	COX10
38680_c0_g2_i1	AtPPT	66	PPT
38680_c0_g2_i2	AtPPT	81	PPT
38680_c0_g2_i3	AtPPT	82	PPT
38680_c0_g2_i4	AtPPT	66	PPT
38680_c0_g2_i5	AtPPT	64	PPT
38680_c0_g2_i7	AtPPT	65	PPT
38680_c0_g2_i9	AtPPT	65	PPT
38680_c0_g3_i2	AtPPT	77	PPT
38680_c0_g3_i3	AtPPT	66	PPT
38680_c0_g3_i4	AtPPT	65	PPT
38680_c0_g3_i5	AtPPT	82	PPT
38680_c0_g3_i6	AtPPT	82	PPT
5590_c0_g1_i1	AtVTE2-1	70	VTE2-1
18296_c0_g1_i1	AtVTE2-1	74	VTE2-1
24714_c0_g1_i1	AtVTE2-1	78	VTE2-1
30109_c0_g1_i1	AtVTE2-1	73	VTE2-1
36048_c2_g1_i1	AtVTE2-1	65	VTE2-1
36048_c2_g1_i4	AtVTE2-1	65	VTE2-1
36048_c3_g1_i1	AtVTE2-1	64	VTE2-1
36048_c3_g1_i2	AtVTE2-1	65	VTE2-1
17595_c0_g2_i1	AtVTE2-2	85	VTE2-2
29982_c1_g1_i1	AtVTE2-2	69	VTE2-2
29982_c1_g1_i2	AtVTE2-2	82	VTE2-2
29982_c1_g1_i3	AtVTE2-2	82	VTE2-2
34721_c0_g1_i1	AtVTE2-2	78	VTE2-2
34721_c0_g1_i3	AtVTE2-2	83	VTE2-2
34721_c0_g1_i5	AtVTE2-2	82	VTE2-2

The UbiA superfamily was classified into 69 contigs, each of which was further annotated as a contig of unknown function or U6DT (a), or one involved in primary metabolism (b), based on its amino acid identity to the eight queries. Contigs showing the highest amino acid identities to FcPT1a or FcPT1b were grouped into (a) and those showing the highest amino acid identities with those of the six primary metabolite PTs grouped into (a) or (b) based on whether their identities were below or over the calculated threshold values. Thresholds were set for individual queries at the amino acid levels between them and their orthologous proteins from rice, *i.e.*, 63%, 66%, 58%, 53%, 76%, and 55% for VTE2-1, VTE2-2, PPT, ABC4, ATG4, and COX10, respectively. Asterisks indicate contigs with low ratios relative to fruit latex in Fig. 6. Contigs predicted to possess U6DT or unknown functions are highlighted with red or orange letters, respectively.

New Phytologist

Table S4 PT genes used for gene structure analysis

Database	Gene	Chromosome or scaffold number	Position (bp)	Strand
NCBI	*FcPT1*	BDEM01000717.1	9,576 - 12,500	+
	FcVTE2-1	BDEM01000926.1	64,132 - 69,407	-
	FcVTE2-2	BDEM01000270.1	169,550 - 173,963	-
Phytozome	*AtVTE2-1*	Chr2	8,207,491 - 8,210,047	+
	AtVTE2-2	Chr3	3,780,041 - 3,782,880	-
	AtrVTE2-1	scaffold00019	5,097,620 - 5,105,062	+
	AtrVTE2-2	scaffold00068	2,058,132 - 2,080,007	-
	CrVTE2-2	chromosome_6	5,379,781 - 5,382,312	+
	CsVTE2-1	scaffold_19	1,277,349 - 1,279,496	-
	DcVTE2-1	DCARv2_Chr5	25,417,526 - 25,423,710	+
	DcVTE2-2	DCARv2_Chr4	28,300,773 - 28,306,037	+
	GmVTE2-1	Chr13	21,299,292 - 21,304,948	+
	GmVTE2-2	Chr02	25,883,711 - 25,891,047	+
	MpVTE2-1	scaffold_42	807,856 - 812,844	-
	MpVTE2-2	scaffold_82	401,183 - 402,409	-
	OsVTE2-1	Chr6	27,087,681 - 27,092,497	+
	OsVTE2-2	Chr7	23,302,619 - 23,308,978	+
	SmVTE2-1	scaffold_68	361,540 - 363,268	-
	SmVTE2-2	scaffold_40	1,396,141 - 1,397,796	+
	ZmVTE2-1	9	107,429,884 - 107,438,797	-
	ZmVTE2-2	2	207,511,380 - 207,526,670	+
MorusDB	*MnIDT*	KE343433	360,147 - 363,899	-

References in the supporting information

Chase MW, Christenhusz MJM, Fay MF, Byng JW, Judd WS, Soltis DE, Mabberley DJ, Sennikov AN, Soltis PS, Stevens PF. 2016. An update of the Angiosperm Phylogeny Group classification for the orders and families of flowering plants: APG IV. *Botanical Journal of the Linnean Society* **181**: 1–20.

Kawai Y, Ono E, Mizutani M. 2014. Evolution and diversity of the 2–oxoglutarate-dependent dioxygenase superfamily in plants. *The Plant Journal* **78**: 328–343.

Kitajima S, Aoki W, Shibata D, Nakajima D, Sakurai N, Yazaki K, Munakata R, Taira T, Kobayashi M, Aburaya S et al. 2018. Comparative multi-omics analysis reveals diverse latex-based defense strategies against pests among latex-producing organs of the fig tree (*Ficus carica*). *Planta* **247**: 1423–1438.

Mori K, Shirasawa K, Nogata H, Hirata C, Tashiro K, Habu T, Kim S, Himeno S, Kuhara S, Ikegami H. 2017. Identification of *RAN1* orthologue associated with sex determination through whole genome sequencing analysis in fig (*Ficus carica* L.). *Scientific reports* **7**: 41124.

Murray RDH, Mendez J, Brown SA. 1982. *The natural coumarins*. Wiley & Sons, New York.

Simons R, Vincken JP, Bakx EJ, Verbruggen MA, Gruppen H. 2009. A rapid screening method for prenylated flavonoids with ultra-high-performance liquid chromatography/electrospray ionisation mass spectrometry in licorice root extracts. *Rapid Communications in Mass Spectrometry* **23**: 3083–3093.

Vanholme R, Sundin L, Seetso KC, Kim H, Liu X, Li J, De Meester B, Hoengenaert L, Goeminne G, Morreel K et al. 2019. COSY catalyses *trans-cis* isomerization and lactonization in the biosynthesis of coumarins. *Nature Plants* **5**: 1066–1075.
